# Reduced-order modeling of solute transport within physiologically realistic solid tumor microenvironment

**DOI:** 10.3389/fphar.2026.1746751

**Published:** 2026-03-10

**Authors:** Mohammad Mehedi Hasan Akash, Mohammad Yeasin, Shima Mahmoudirad, Redowan A. Niloy, Jiyan Mohammad, Katie Reindl, Anupam Pandey, Saikat Basu

**Affiliations:** 1 Department of Mechanical Engineering, South Dakota State University, Brookings, SD, United States; 2 Department of Mechanical Engineering, Florida State University, Tallahassee, FL, United States; 3 Department of Mechanical and Aerospace Engineering, Syracuse University, Syracuse, NY, United States; 4 Department of Aerospace and Mechanical Engineering, University of Notre Dame, Notre Dame, IN, United States; 5 Department of Biological Sciences, North Dakota State University, Fargo, ND, United States

**Keywords:** electrohydrodynamics (EHD), glycocalyx, multiphase simulation, plasma perfusion, reduced order biomimetic modeling, reverse advection-diffusion (RAD) model

## Abstract

**Introduction:**

Solid tumors are characterized by densely packed extracellular matrices and limited vascularization, creating significant resistance to both diffusive and convective transport. Tumor growth depends on complex flow–structure interactions across multiple scales, while vascular abnormalities and enhanced permeability elevate interstitial pressure in the tumor microenvironment.

**Methods:**

In this study, we developed an integrated computational framework with a theoretical modeling framework that couples three phase, viscous-laminar, transient simulations of glycocalyx-patched tumor vessel-resolving plasma, red blood cells (RBCs), and white blood cells (WBCs) and tracking their volume fractions with a calibrated reverse advection-diffusion (RAD) model for intratumoral plasma transport. The reduced-order tumor microenvironment model incorporates electrohydrodynamic (EHD) force at the tumor vessel wall via glycocalyx patches on the luminal surface.

**Results:**

At the fenestra, EHD increases inlet plasma intensity relative to a non-EHD framework across all 15 numerical models (means: 0.576 non-EHD vs. 0.722 EHD; gain 25.34% ). Numerical simulations of plasma perfusion in both the tumor ECM domain and a microfluidic benchmark exhibit two-stage kinetics, with an initial advection-dominated regime. The RAD model reproduces this behavior and, after temporal calibration, matches the observed propagation.

**Discussion:**

By using fully resolved, EHD-inclusive multiphase CFD simulations to calibrate a reduced-order RAD model parameterized by measurable geometric features, we bridge the gap between classical Darcy–Starling perfusion models and fully resolved CFD. The resulting framework provides a tractable mechanism-grounded tool for quantifying plasma progression in dense solid tumors and for establishing the baseline transport capacity of the tumor extracellular matrix, independent of solute-specific biochemical properties.

## 1 Introduction

Solute transport in solid tumors is fundamentally governed by the structural and mechanical properties of the surrounding tissue (Gullino, 1980; Sefidgar et al., 2014). Irregular vascular architecture, dense stromal packing, and elevated interstitial resistance disrupt flow pathways and reduce transport efficiency (Vaupel et al., 1990; Jain et al., 2014; Koumoutsakos et al., 2013; Stylianopoulos et al., 2018; Jiang et al., 2020). These conditions create spatial variability in pressure-driven and diffusive mechanisms, producing highly non-uniform intratumoral distributions. Despite the enhanced permeability and retention effect often reported for nanoparticle delivery (Maeda, 2012), perfusion in tumors can be much lower than in surrounding healthy tissues due to vascular constriction and abnormal leakiness into the extracellular space (Stylianopoulos and Jain, 2013).

Solid tumors, which constitute the majority of cases, present as noncystic masses categorized as benign or malignant (Gavhane et al., 2011; Rahman et al., 2023) and exhibit high resistance to diffusion (Jain, 1996; Jain, 1998) with limited blood and lymphatic drainage that impedes advection (Chim and Mikos, 2018). Accurate, mechanics-based quantification of spatiotemporal solute distributions is therefore essential for clinical decision-making (d’Esposito et al., 2018), as flow–structure interactions and vascular leakiness elevate interstitial pressure and vary across patient-specific tumor topography (Soltani and Chen, 2011; Zhang et al., 2013). Beyond hydrodynamics, electrohydrodynamic (EHD) introduces electrical body forces that can bias plasma transport. *In vivo*, the endothelial glycocalyx presents spatially patched and heterogeneous surface charge distributions that can generate localized EHD responses at the vessel wall (Weinbaum et al., 2007; Huilgo and Phan-Thien, 1997; Sumets et al., 2018; Teodoro et al., 2024). These forces act directly on the plasma phase and can therefore influence transport behavior in regions where plasma extravasation is initiated, such as near fenestral openings. In confined tumor microvascular geometries, localized electric body forces may become comparable to hydrodynamic stresses at the vessel wall, motivating the need for explicit vessel-scale modeling of EHD effects. Resolving these interactions is essential to capture the mechanistic “gatekeeping” behavior of the vessel wall, which lies outside the scope of purely pressure-driven descriptions of transvascular transport. Blood flow is multiphase and non-Newtonian: plasma with suspended RBCs and WBCs forms a heterogeneous mixture whose collective effects give rise to shear-dependent rheology and dispersion behavior (Merrill et al., 1963; Baskurt and Meiselman, 2003). Two phase hemodynamics models (Jung et al., 2006a) improve over single-phase assumptions yet remain incomplete; three-phase formulations (Akash et al., 2023) better capture particulate interactions and motivate coupling geometry and biology in a predictive framework. Prior multiphase–EHD studies (Rahmat, 2017) provide valuable numerical background but typically do not incorporate explicit three-phase blood or patched glycocalyx charge (Kang et al., 2018). In parallel, classical mean-transport models (Darcy’s law (Baish et al., 2011); Starling’s equation (Sugihara-Seki and Fu, 2005)) remain useful at the tissue scale but overlook key features of tumor hemodynamics, including non-Newtonian, particulate rheology, and geometry-specific microstructure. At the tissue scale, Darcy–Starling models provide an effective mean description of transport but treat the plasma flux across the vessel wall as a prescribed quantity. In contrast, the present study interrogates the vessel-wall-scale physics that govern the initiation of plasma entry into the interstitium, including localized electrohydrodynamic forcing and multiphase interactions near fenestral openings. Likewise, single-phase computational fluid dynamics (CFD), which treats blood as a homogeneous continuum, cannot resolve the interactive dynamics and spatial distribution of suspended particulate matter (Womersley, 1957; Attinger, 1964; Srivastava, 2007). Recent computational studies have explored tumor transport using porous-media, discrete–continuum, and hybrid multiscale formulations, providing valuable insight into pressure-driven perfusion and solute redistribution in complex vascularized tissues (Vavourakis et al., 2018; Sweeney et al., 2024; Wirthl et al., 2020). Complementary review articles have further emphasized the importance of multiscale coupling, structural heterogeneity, and transport bottlenecks in cancer modeling (Mahesh et al., 2022; Budhwani et al., 2022; Lorenzo et al., 2024; Hadjicharalambous et al., 2021). However, these approaches generally prescribe vessel-wall transport phenomenologically and do not explicitly resolve electrohydrodynamic effects arising from heterogeneous glycocalyx charge at fenestral interfaces. The present study addresses this gap by directly coupling vessel-resolved, EHD-inclusive multiphase simulations to downstream ECM transport models.

Building on these advances and remaining gaps, we develop a three-phase simulation framework that explicitly resolves blood mechanics and glycocalyx-mediated EHD inside tumor vessels to mechanistically quantify—and, under controlled conditions, enhance plasma ingress into the tumor extracellular matrix (ECM) in a biologically faithful manner. Building on these high-fidelity simulations, we introduce a CFD-informed reverse advection-diffusion (RAD) model: an advection–diffusion framework whose boundary data and closures are calibrated from the resolved vessel physics and whose coefficients are expressed in terms of measurable geometric descriptors such as stromal packing fraction (Sefidgar et al., 2014; Sefidgar et al., 2015; Soltani and Chen, 2011). Here, “reverse” denotes a transport regime in which the net progression of plasma-borne species within the ECM region is biased in the opposite direction to the expected forward perfusion direction predicted by pressure-driven Darcy flow, due to geometric obstruction and elevated interstitial resistance. The fundamental objective of this study is to quantify the mechanistic barriers governing plasma ingress from a microvessel into the tumor extracellular matrix (ECM). While plasma transport ultimately enables the delivery of nutrients and other carried species *in vivo*, the present work focuses specifically on the fluid-mechanical behavior of the carrier phase itself. We emphasize plasma because its perfusion kinetics represent a key transport-limiting step in dense, poorly vascularized tumor tissue. Within this framework, plasma volume fraction computed in CFD, dye-front advancement observed in microfluidic experiments, and solute fields obtained from reduced-order transport modeling are treated as complementary, scale-dependent descriptors of the same underlying physical process: the spatiotemporal wetting and saturation of the tumor interstitium. By unifying these metrics, the model is designed to interrogate how localized vessel-scale mechanisms, such as electrohydrodynamic (EHD) forcing, bias the initiation and depth of interstitial plasma transport. Our aim is to retain the mechanistic fidelity of vessel-resolved three-phase CFD—including localized EHD at patched glycocalyx segments–while bridging the gap between classical Darcy–Starling formulations and modern fluid dynamics by embedding these vessel-scale effects into a reduced-order transport model. This motivates the following questions:

Q1. As a marker of enhanced biological realism, how would the inclusion of electrohydrodynamic (EHD) effects change the plasma extravasation across the vessel wall at the fenestra, relative to a non-EHD baseline?

Q2. As a parametric tool, could we set up a simplified, reduced-order reverse advection-diffusion (RAD), informed by vessel-scale CFD-derived inlet conditions, to reproduce the spatiotemporal progression of plasma transport in the ECM without explicitly resolving multiphase or electrohydrodynamic effects?

To address these questions, we first perform three phase CFD of tumor-vessel flow with EHD localized to the vessel domain to quantify changes in plasma-perfusion metrics relative to a non-EHD baseline; we then develop and calibrate the RAD model to express penetration and advection diffusion transitions explicitly in terms of packing fraction and geometry, thereby bridging the gap between classical mean-transport descriptions and fully resolved CFD. Finally, we benchmark the model’s key predictions (front advance, wetted-area scaling, and transition time) against complementary microfluidic validation.

### 1.1 Extension of prior work and current scope

The present study builds directly upon our previous multiphase modeling framework for tumor microvessels and plasma transport in the extracellular matrix (ECM) (Akash et al., 2023). In that earlier work, blood flow within the tumor vessel was modeled as a three-phase system (plasma–RBC–WBC), and plasma transport in the ECM was described using a two-phase formulation, focusing on fenestra-mediated plasma leakage under purely hydrodynamic conditions. The current study retains this multiphase foundation but introduces several new physical, methodological, and validation components that substantially extend the scope and insight of the framework. *Electrohydrodynamic (EHD) physics:* The primary physical extension is the inclusion of electrohydrodynamic (EHD) forcing at the vessel wall through glycocalyx-mediated surface charge heterogeneity. This mechanism was not considered in the prior work and enables direct investigation of how localized electrokinetic forces bias plasma ingress at the fenestra. Incorporating EHD reveals a consistent enhancement in plasma perfusion intensity relative to non-EHD conditions, with a mean gain of 25.34% across all cases. *Systematic spatial sensitivity analysis.* Beyond introducing EHD, the present study expands the analysis from a limited set of geometries to an ensemble of N=15 vessel models, generated by translating the fenestra center axially along the vessel over an effective length of 450 μ m. This systematic parametric sweep allows us to quantify how fenestra location modulates EHD-driven plasma transport and to assess the robustness of EHD effects across spatial variability, rather than relying on a single representative configuration. *Experimental validation.* The computational framework is further strengthened through direct benchmarking against complementary microfluidic experiments using 3D-printed biomimetic ECM structures. These experiments validate plasma front advancement and perfusion trends predicted by the simulations, providing experimental grounding that was not present in the prior computational-only study. *Reverse advection-diffusion (RAD) theoretical framework integration:* In addition to the high-fidelity multiphase simulations, the present study introduces a reduced-order reverse advection–diffusion (RAD) model to describe plasma progression within the ECM. The RAD model is not a multiphase formulation and does not solve the Navier–Stokes equations. Instead, it provides a theoretical, continuum description of plasma transport based on an advection–diffusion equation posed on a simplified ECM geometry. The RAD model serves as a computationally efficient surrogate that reproduces the spatiotemporal progression of plasma transport observed in CFD, while deliberately abstracting away detailed fluid mechanics in favor of mechanistic interpretability and parametric exploration.

## 2 Methods

### 2.1 Domain reconstruction

#### 2.1.1 Development of reduced-order tumor vessel

**FIGURE 1:**
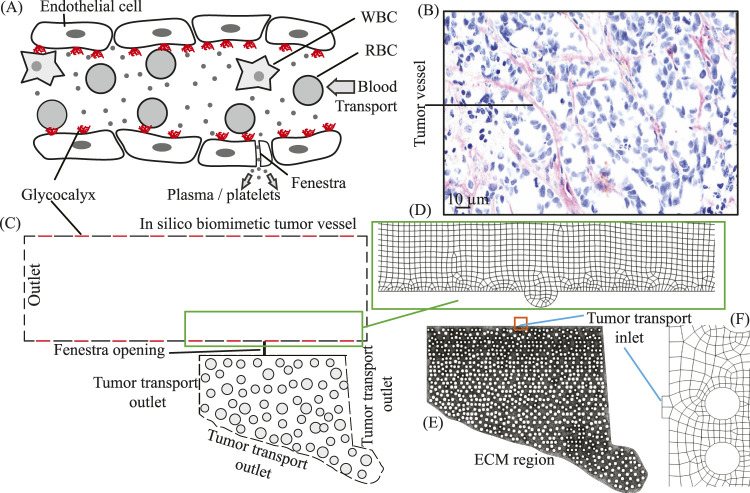
Physiologically inspired schematic and simulation geometry of a tumor vessel coupled to the surrounding tumor ECM domain: **(A)** Endothelial capillary schematic showing the endothelial glycocalyx (red patches), red blood cells (RBCs), white blood cells (WBCs), and a plasma-only fenestra that connects the vessel to the tumor extracellular matrix (ECM). This schematic motivates the reduced-order model; in panel **(C)**, red lines indicate glycocalyx-patched regions where electrohydrodynamic (EHD) forcing is applied, whereas black lines represent non-patched endothelial surfaces with no applied EHD forcing. **(B)** Representative histology of a tumor vessel and a scanned cross-section of a dense pancreatic tumor implanted in mice; this morphology informed the simulated vessel. **(C)** Two dimensional biomimetic domain of the tumor microenvironment, where the reduced-order tumor vessel is connected to the tumor stroma via a fenestra. For numerical simulations, the domain in **(C)** is partitioned into two segments. The modeling strategy restricts the entry of suspended particulates at the fenestra by selecting a fenestra diameter smaller than typical particle sizes. **(D–F)** The two segments of the test geometry with their corresponding meshes: **(D)** a portion of the tumor vessel showing the circular wall depression that coincides with the fenestra location; **(E)** the ECM region with the fenestra (tumor-transport inlet); and **(F)** a zoomed-in view of the fenestra region with finer mesh elements. Plasma pressure and velocity fields extracted at the fenestra center (panel D) from the vessel-scale simulations define the inlet boundary conditions for the ECM transport domain (panel E), providing a one-way coupling from the intravascular to the extravascular space. Additional details of the ECM geometry are provided in Section 2.1.2.

**FIGURE 2:**
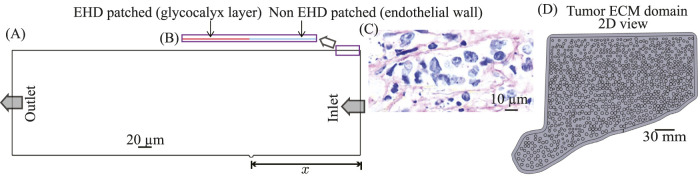
Reduced-order tumor vessel and ECM geometries used in the present numerical framework **(A)** Reduced-order tumor vessel with inlet (right) and outlet (left). The x marks the admissible interval for the fenestra center used in the parametric sweep; varying x changes the model index (Models 1–15 per Equation 1). **(B)** Zoomed view from the actual reduced-order CAD model showing alternating surface segments red line shows glycocalyx-patched regions where localized electrohydrodynamic (EHD) forcing is applied on the luminal surface of the tumor vessel; while blue line shows non-patched endothelial wall regions with no EHD forcing on the luminal surface of the tumor vessel (implementation details in Section 2.2.1). **(C)** Representative H&E histology image of a tumor slice illustrating irregular microvascular morphology; this image is shown for physiological context only and is not used to define the simulation geometry. **(D)** Planar (2D) surrogate of the tumor ECM domain used for transport simulations; populated with circular fiber bundles to achieve the prescribed packing fraction. The ECM domain is coupled to the vessel only through the fenestra inlet boundary conditions derived from vessel-scale simulations.

To interrogate electrohydrodynamic (EHD)-driven changes in plasma perfusion, we employed a biomimetic tumor microvessel coupled to an extracellular matrix (ECM) domain (Figure 1C). The reduced-order vessel geometry follows the configuration in our prior work and its validated *in silico* capillary model (Akash et al., 2023; Akash et al., 2024; Akash et al., 2021): a 500 μ m-long two dimensional planar channel of height 150 μ m with a single fenestra, modeled as a rounded wall depression, which serves as the sole plasma-ECM exchange interface. The vessel wall contains a micron-scale rounded endothelial depression that defines the geometric access region for plasma extravasation. This depression (Figure 1D) is 2.6 μm deep and 5.2 μm in diameter, sized to permit plasma entry while excluding larger formed elements such as red blood cells (RBCs, 7– 8 μm ) (Nithiarasu, 2022). It is designed to fully contain the sub-micron fenestral opening that mediates plasma transport into the extracellular matrix. The effective fenestra (transport aperture) has a characteristic streamwise length of approximately 1.0 μ m (Figure 1E). The larger depression ensures that the entire fenestral region is geometrically resolved within the vessel wall and that particulate exclusion (e.g., RBCs) is enforced upstream of the transport aperture. A single fenestra is modeled as a representative plasma–ECM exchange site, under the assumption that fenestrae along tumor capillaries act as spatially localized and largely independent transport units at the scales considered. This representation enables isolation of local plasma entry mechanics without introducing additional geometric repetition, while preserving the governing transport physics at the vessel–ECM interface. Endothelial-scale context, including endothelial cells, glycocalyx, RBCs, and white blood cells (WBCs), is illustrated in Figure 1A, and the near-fenestra mesh is shown in Figure 1D. To represent heterogeneous endothelial surface properties that mediate EHD, we applied wall-attached glycocalyx patches (Figure 2B) with fixed streamwise extent of 0.5 μm Dull and Hahn (2022); Weinbaum et al. (2021). Patches were distributed in a randomized, nonperiodic pattern (some adjacent, others separated by a single gap) and held fixed across all cases for reproducibility. Numerically, patched regions were implemented via a user-defined forcing term localized to wall-adjacent control volumes, adding an electric body force consistent with the prescribed patched surface charge (details in Section 2.2.1). In the schematic and layout, red wall segments denote patched, glycocalyx-bearing regions where EHD forcing is applied (Figures 1A,C), whereas black wall segments in Figure 1C indicate non-patched wall with no EHD forcing.

Sensitivity of perfusion to the location of plasma entry was probed by generating N=15 geometries in which the fenestra center was shifted axially along the vessel while keeping fenestra shape and the patch pattern unchanged (Figure 2A). With 25 μm clearance zones at inlet and outlet, admissible centers span [25, 475] μm . We define

$$L_{\text{center}}^{\left(i\right)}=25\;\mu \text{m}+\left(i-1\right)\;\Delta L,\;\Delta L=\frac{450\;\mu \text{m}}{N-1}=32.1429\;\mu \text{m},\;i=1,\ldots ,15,$$ (1)

which places one non-overlapping fenestra per configuration over the effective length $L_{\text{eff}}=450\;\mu \text{m}$ . The first admissible center at $L_{\text{center}}^{\left(1\right)}=25\;\mu \text{m}$ is Model 1, and the sequence proceeds in steps of ΔL=32.1429 μm so that Model 15 corresponds to $L_{\text{center}}^{\left(15\right)}=475\;\mu \text{m}$ .

All geometries were constructed and meshed in ANSYS Workbench 2023 R1 using DesignModeler (DM) for CAD and the integrated mesher (ANSYS Inc., Canonsburg, Pennsylvania). Because the computational domain is two-dimensional (Figures 1C–F), unstructured triangular meshes were employed, with local refinement around the fenestra and patched-wall regions to resolve steep pressure/velocity gradients while keeping the far field economical.

#### 2.1.2 Development of the tumor extracellular matrix (ECM) domain

We constructed the adjacent ECM region and populated it to achieve a prescribed in-plane packing fraction. The ECM geometry was designed to match a representative solid packing fraction of 0.30, supported by quantitative color-decomposition analysis of tumor cross-sectional histology in our prior work (local range 0.23–0.41) (Akash et al., 2023) and by independent MRI measurements reporting absolute tumor interstitial fluid volume fractions of approximately 27.5%, consistent with a comparably dense solid ECM environment (Kim et al., 2004). In the ECM model, this packing fraction was implemented by representing stromal crowding by $N_{\text{fib}}=870$ (Figure 2D) circular bundles placed within the ECM such that

$$\phi \equiv \frac{A_{\text{fib}}}{A_{\text{dom}}}=0.30,$$ (2)

with a target mean bundle diameter $\bar{d}\approx 3.87\;\mu m$ (range 3.0– 4.5 μm ). Here $A_{\text{fib}}$ is the total fiber cross-sectional area and $A_{\text{dom}}$ is the ECM domain area (Equation 2). Fixing ϕ sets the porosity ε=1−ϕ , which enters the transport closures for the effective diffusivity $D_{\text{eff}}$ (via tortuosity) and the hydraulic permeability k , thereby limiting both diffusive penetration and pressure-driven perfusion. The imposed fiber field was checked against orientation statistics from the same segmentation workflow and against reported stromal-alignment trends in tumor tissues (Eekhoff and Lake, 2020). Coupling to the vessel is applied along the extracted boundary locus at the fenestra by prescribing the plasma-field inlet (and associated flux) from the vessel simulation; this enforces a smooth interface without explicitly meshing the vessel solid within the ECM grid. The two dimensional ECM domain (Figure 2D) was meshed with approximately $5.9\times 10^{4}$ unstructured triangular elements (see also Figure 1E), with local refinement near the vessel–ECM interface. Near the boundary locus corresponding to the fenestra, a characteristic length scale $a_{\text{fen}}=0.1\;\mu m$ was used in the near-wall mass-transfer closure and scaled locally with the extracted wall geometry. In the simulations, the multiphase transport calculation in the vessel yields the plasma volume fraction at the artificial circular depression (fenestra), which is then used as the inlet condition for the subsequent ECM computation (Figure 1E), where intratumoral plasma transport is tracked.

### 2.2 Simulation setup

#### 2.2.1 Simulation method for blood flow inside the tumor vessel

We model the *in silico* transport of blood within tumor vessels as an unsteady, viscous–laminar, two dimensional (planar) flow. Numerical solutions are obtained in ANSYS Inc., 2023 R1 using its Eulerian multiphase model (no custom inter-particle equations). Plasma is the primary (continuous) phase; red blood cells (RBCs) and white blood cells (WBCs) are treated as two dispersed phases. The formulation follows the classical multiphase CFD framework (Gidaspow, 1994; Anderson and Jackson, 1967) and is appropriate for *in vivo* hematocrits of 30%–55% (Jung et al., 2006a). Closures (mixture shear-thinning, interphase drag, agglomeration shape factor) follow (Akash et al., 2023). The governing conservation equations for the tumor-vessel simulations, together with the associated electrohydrodynamic and rheological closure relations, are given in Equations 3–11 and are solved numerically.

Let k∈{p,r,w} index plasma (p) , RBCs (r) , and WBCs (w) , with volume fractions $\alpha_{k}$ and velocities $u_{k}=\left(u_{k},v_{k}\right)$ in 2D, and let $\nabla =\left(\partial_{x},\partial_{y}\right)$ . Interpenetrating phases satisfy

$$\alpha_{p}+\alpha_{r}+\alpha_{w}=1,\;0\leq \alpha_{k}\leq 1,$$ (3)

and, under phasewise incompressibility,

$$\partial_{t}\alpha_{k}+\nabla \;\cdot \left(\alpha_{k}\;u_{k}\right)=0,\;k\in \left\{p,r,w\right\}.$$ (4)

Phase momenta evolve as

$$\partial_{t}\left(\alpha_{k}\rho_{k}\;u_{k}\right)+\nabla \;\cdot \left(\alpha_{k}\rho_{k}\;u_{k}⊗u_{k}\right)=-\alpha_{k}\nabla p+\nabla \;\cdot \left(\alpha_{k}\;\tau_{k}\right)+\alpha_{k}\rho_{k}\;g+\underset{j\neq k}{\sum }M_{kj}+\alpha_{k}\;\chi_{\text{tumor}}\;f_{E}^{\left(k\right)},$$ (5)

with a common mixture pressure p , viscous stress $\tau_{k}$ , and interphase exchange $M_{kj}$ satisfying $\sum_{k}\sum_{j\neq k}M_{kj}=0$ . Phase densities used here are $\rho_{p}=1030\;kg/m^{3}$ , $\rho_{r}=1100\;kg/m^{3}$ , $\rho_{w}=1080\;kg/m^{3}$ (Nithiarasu, 2022; Schwartz and Conley, 2020). The indicator $\chi_{\text{tumor}}\in \left\{0,1\right\}$ activates electrohydrodynamic (EHD) forcing only in the tumor–vessel subdomain.

The electroquasistatic potential satisfies

$$-\nabla \;\cdot \left(\varepsilon \left(x\right)\;\nabla \phi \right)=\rho_{e},\;E=-\nabla \phi =\left(E_{x},E_{y}\right)=\left(-\partial_{x}\phi ,-\partial_{y}\phi \right),$$ (6)

where $\varepsilon =\epsilon_{0}\epsilon_{r}$ is the permittivity (F/m) , $\epsilon_{0}$ is the vacuum permittivity, $\epsilon_{r}$ is the case-specific relative permittivity, and $\rho_{e}$ is the free charge density $\left(C/m^{3}\right)$ . The Kelvin (electrohydrodynamic) body force $\left(f_{E}\right)$ added to Equation 5 is

$$f_{E}=\rho_{e}\;E-\frac{1}{2}\;\left(\nabla \varepsilon \right)\|E{\|}^{2},$$ (7)

as in continuum EHD theory (Landau et al., 2013; Saville, 1997; Castellanos et al., 2003). In this study ε is treated as spatially uniform unless otherwise noted, so $f_{E}=\rho_{e}\;E$ . The Fluent UDF applies the phase-aggregated force components (where E is the electric potential)

$$\begin{array}{cc} F_{Ex} & =\left(\rho_{e,p}+\rho_{e,r}+\rho_{e,w}\right)\;E_{x}+\zeta \;\epsilon_{0}\;\epsilon_{r}\;E_{x},\\ F_{Ey} & =\left(\rho_{e,p}+\rho_{e,r}+\rho_{e,w}\right)\;E_{y}+\zeta \;\epsilon_{0}\;\epsilon_{r}\;E_{y}, \end{array}$$ (8)

where $\rho_{e,k}$ are phase charge densities and $\rho_{e}=\sum_{k}\rho_{e,k}$ ; ζ is the zeta potential applied on EHD-active glycocalyx patches. Electrical properties used in Equations 6–8 are: ζ=−0.01 V (Squires and Bazant, 2006); $\epsilon_{0}=8.854\times 10^{-12}\;F/m$ ; phase conductivities $\sigma_{p}=1.2\;S/m$ (Pauly and Schwan, 1966), $\sigma_{r}=0.52\;S/m$ (Zhbanov and Yang, 2015), $\sigma_{w}=0.64\;S/m$ (Yang et al., 1999); and a case-specific $\epsilon_{r}$ stated with each EHD case.

The non-Newtonian shear-thinning behavior of blood is introduced in this numerical framework as a phenomenological mixture-level closure calibrated to experimental rheology, rather than as an emergent consequence of resolved red blood cell deformation or aggregation. While RBCs and WBCs are modeled as dispersed Eulerian phases to capture phase segregation and transport heterogeneity, the present formulation does not resolve cell-scale mechanics required for self-consistent rheological emergence. Specifically, because the Eulerian dispersed phases are treated as interpenetrating continua without explicit cell-membrane deformation or aggregation dynamics, phase kinematics are decoupled from the constitutive mixture-level stress response, thereby necessitating the use of an empirical rheological closure. The blood’s shear-thinning behavior is represented by (Jung and Hassanein, 2008; Jung et al., 2006b). With hematocrit $\alpha_{r}$ (RBC volume fraction) and scalar shear rate $\dot{\gamma }$ ,

$$\mu_{\text{mix}}=\left(\frac{\alpha_{r}\;\eta_{\text{RBC}}+\alpha_{p}\;\eta_{\text{plasma}}+\alpha_{w}\;\eta_{\text{WBC}}}{\eta_{\text{plasma}}}\right)=a\;{\left[1+{\left(\tau \dot{\gamma }\right)}^{2}\right]}^{\left(b-1\right)/2},\;\tau =0.110\;sec,$$ (9)

where $\mu_{\text{mix}}$ is the mixture dynamic viscosity (Pa ⋅ s), $\eta_{\text{plasma}}=0.001\;kg/\left(m\cdot s\right)$ (Nithiarasu, 2022), and we take $\eta_{\text{WBC}}=0.011\;kg/\left(m\cdot s\right)$ (Schwartz and Conley, 2020). The effective RBC contribution is captured through the hematocrit-dependent mixture law; no separate constant $\eta_{\text{RBC}}$ is prescribed. The coefficients $a\left(\alpha_{r}\right)$ and $b\left(\alpha_{r}\right)$ are dimensionless Carreau–Yasuda–type fits to $\alpha_{r}$ :

$$\begin{array}{cc} a & =122.28\;\alpha_{r}^{3}-51.213\;\alpha_{r}^{2}+16.305\;\alpha_{r}+1,\\ b & =0.8092\;\alpha_{r}^{3}-0.8246\;\alpha_{r}^{2}-0.3503\;\alpha_{r}+1, \end{array}$$ (10)

and, for $\dot{\gamma }<6\;{sec}^{-1}$ ,

$$\begin{array}{cc} a & =70.782\;\alpha_{r}^{3}-22.454\;\alpha_{r}^{2}+9.7193\;\alpha_{r}+1,\\ \frac{b-1}{k} & =-0.8913\;\alpha_{r}^{3}+2.0679\;\alpha_{r}^{2}-1.7814\;\alpha_{r},\;k=\frac{\ln\left(\ln\dot{\gamma }\right)}{\ln\left(\dot{\gamma }\right)}, \end{array}$$ (11)

where k is the (dimensionless) Yasuda exponent. Interphase drag follows Schiller–Naumann (Naumann and Schiller, 1935); RBC agglomeration enters via the dynamic shape factor

$$\xi =\left\{\begin{array}{cc} 1.5\;{\left[1+{\left(\tau \dot{\gamma }\right)}^{2}\right]}^{0.058697}, & \dot{\gamma }\leq 300\;{\text{sec}}^{-1},\\ 1, & \text{otherwise}, \end{array}\right.$$

while WBCs use ξ=1 . Characteristic cell sizes used in closures are $d_{\text{RBC}}=7\;\mu m$ and $d_{\text{WBC}}=14\;\mu m$ (Nithiarasu, 2022; Schwartz and Conley, 2020). The transport equations inside tumor vessel were discretized using the finite-volume method implemented in ANSYS FLUENT. An implicit Eulerian multiphase formulation was employed, with pressure–velocity coupling handled via the phase-coupled SIMPLE algorithm. Spatial discretization used least-squares cell-based gradient reconstruction, second-order schemes for pressure, and second-order upwind discretization for convective terms. Temporal integration was performed using a second-order implicit transient formulation with a constant time step $\Delta t=1.0\times 10^{-4}$ s. Across the ensemble of 15 tumor-vessel simulations, the Courant–Friedrichs–Lewy (CFL) number remained below unity, ensuring numerical stability and temporal convergence throughout the simulation duration.

*Boundary conditions for the tumor-vessel calculation are*: pressure inlet $p_{\text{in}}=3325\;Pa$ and pressure outlet $p_{\text{out}}=2128\;Pa$ (Zhao et al., 2007). At the outlet, the backflow volume fraction is set to RBC =1 and WBC =0 to prevent unphysical WBC re-entry while remaining consistent with the dispersed-phase specification. Electric potentials ϕ are applied only on tumor–vessel boundaries where EHD is active; elsewhere $\partial_{n}\phi =0$ . Time integration employed a constant time step $\Delta t=1.0\times 10^{-4}\;sec$ ; each vessel case was advanced for 2000 iterations, matching the ECM simulation, with convergence monitored via mass and momentum residuals and outlet flux balances. Computations were run in parallel on four Xeon cores at 3.1 GHz. From each of the fifteen vessel geometries, plasma pressure and velocity were sampled at the fenestra-center location (wall depression); the arithmetic averages of these fields define the inlet data subsequently used in the ECM (2D) plasma-perfusion simulation.

##### 2.2.1.1 Mesh refinement study and numerical stability

A systematic mesh-refinement study was performed (see Table 1) for the tumor-vessel domain to verify spatial convergence of the multiphase CFD simulations and to ensure that the reported plasma perfusion metrics are independent of discretization resolution. The refinement analysis focused on domain-averaged pressure and velocity, which directly influence plasma transport and extravasation near the fenestral interface. Element size was progressively reduced while maintaining identical boundary conditions and solver settings. Between the 1.00 μm and 0.76 μm meshes, the change in domain-averaged pressure is 0.22 Pa (approximately 0.010% relative to 2180 Pa ), and the change in average velocity magnitude is $1.0\times 10^{-7}\text{ m/s}$ (approximately 0.29% relative to $3.43\times 10^{-5}\text{ m/s}$ ). These sub-percent variations indicate spatial convergence of the numerical solution. Given the marginal accuracy gain relative to the increased computational cost associated with further refinement, the 1.00 μm mesh (77k elements) was selected for all 15 vessel geometries to ensure consistent resolution across the parametric ensemble. Temporal discretization employed a fully implicit second-order scheme with a constant time step of $\Delta t=1.0\times 10^{-4}$ sec. The resulting Courant–Friedrichs–Lewy (CFL) number remained below unity across all vessel realizations, ensuring numerical stability and temporal convergence.

**TABLE 1:** Mesh-refinement study: domain-averaged pressure and velocity. Differences between the 1.00 μm mesh and the finer 0.76 μm mesh are negligible, indicating spatial convergence.

Element size ( μ m)	Total elements	$P_{\text{avg}}$ (Pa)	$V_{\text{avg}}$ (m/s)
5.0	8k	2193.67	$3.1\times 10^{-5}$
3.0	19k	2180.66	$4.3\times 10^{-5}$
1.00	77k	2180.26	$3.43\times 10^{-5}$
0.76	80k	2180.48	$3.44\times 10^{-5}$

#### 2.2.2 Simulation method for plasma perfusion inside the ECM tumor region

Transport within the tumor extracellular matrix (ECM) domain was modeled as an unsteady, viscous–laminar, two-dimensional two-phase flow using the Eulerian multiphase formulation in ANSYS Inc., 2023 R1. A shared pressure field is solved while phasewise continuity and momentum equations advance the primary (plasma) and secondary (air) phases. Interphase coupling is represented through drag exchange terms with coefficients evaluated from the local Reynolds number, consistent with the Eulerian framework (Afolabi and Lee, 2014). Electrohydrodynamic forcing is not applied in the ECM calculation (EHD is restricted to the vessel domain), so the momentum balances here contain only hydrodynamic and interphase exchange terms. The ECM simulations consider plasma as the invading liquid phase and air as a displaced secondary phase, enabling explicit tracking of the advancing plasma front. For each phase k∈{p,a} (plasma p and air a ), volume fractions $\alpha_{k}$ satisfy the constraint

$$\alpha_{p}+\alpha_{a}=1,$$ (12)

and evolve according to the phase continuity equations

$$\partial_{t}\alpha_{k}+\nabla \cdot \left(\alpha_{k}u_{k}\right)=0.$$ (13)

The phase momentum equations are solved in mixture form,

$$\partial_{t}\left(\rho u\right)+\nabla \cdot \left(\rho u⊗u\right)=-\nabla p+\nabla \cdot \tau +\rho g,$$ (14)

where u is the mixture velocity, ρ is the volume-fraction-weighted mixture density, and ***τ*** denotes the viscous stress tensor (Equations 12-14). Interphase momentum exchange is captured implicitly through the Eulerian multiphase closure implemented in FLUENT (ANSYS Inc., 2023; Ishii and Hibiki, 2010).

The ECM transport equations were discretized using the finite-volume method implemented in ANSYS FLUENT. An implicit Eulerian multiphase formulation was employed, with pressure–velocity coupling handled via the phase-coupled SIMPLE algorithm. Spatial discretization used least-squares cell-based gradient reconstruction, second-order schemes for pressure, and second-order upwind discretization for convective terms. Temporal integration was performed using a second-order implicit transient formulation with a constant time step $\Delta t=1.0\times 10^{-4}$ sec. Throughout the ECM simulations, the Courant–Friedrichs–Lewy (CFL) number remained below unity, indicating stable time stepping for the advective transport. Each ECM simulation was advanced for 2000 time steps, matching the duration of the corresponding tumor-vessel simulations. Convergence at each time step was assessed by monitoring residual reduction for mass and momentum equations, as well as global flux balances at the ECM outlets. All computations were executed in parallel on four Xeon cores at 3.1 GHz with a typical wall-clock time of 3–4 h.

*Geometric coupling between tumor vessel and ECM domains:* the fenestra geometry is explicitly resolved only within the tumor vessel domain, where the curved wall depression serves to localize plasma extrusion while restricting the entry of suspended cellular phases based on size. In contrast, the ECM domain models plasma-only transport and therefore does not require explicit replication of the fenestral curvature. Accordingly, the vessel-to-ECM interface is treated as a transport inlet as a flat sampling plane, rather than a direct geometric continuation of the vessel wall. This planar inlet representation enables consistent mapping of time-resolved pressure and velocity fields from the vessel simulations into the ECM domain while avoiding curvature-induced numerical bias in the downstream transport calculations. This domain partitioning strategy follows our prior validated modeling approach and preserves the relevant transport physics at the vessel–ECM interface without introducing additional geometric constraints.

*Boundary conditions of numerical simulation for plasma perfusion inside the ECM tumor region:* The ECM inlet condition is constructed from the tumor vessel simulations in a time-resolved manner. For each of the N=15 vessel cases, the plasma pressure $p^{\left(i\right)}\left(t_{n}\right)$ and velocity $u^{\left(i\right)}\left(t_{n}\right)$ were sampled at the fenestra-center location at every time step $t_{n}=n\Delta t$ , with Δ t = $1.0\times 10^{-4}\;sec$ . At each $t_{n}$ , the ensemble-averaged inlet fields supplied to the ECM domain are defined as

$${\bar{p}}_{\text{in}}\left(t_{n}\right)=\frac{1}{15}\overset{15}{\underset{i=1}{\sum }}p^{\left(i\right)}\left(t_{n}\right),\;{\bar{u}}_{\text{in}}\left(t_{n}\right)=\frac{1}{15}\overset{15}{\underset{i=1}{\sum }}u^{\left(i\right)}\left(t_{n}\right),$$ (15)

thereby preserving the transient vessel-scale dynamics in the downstream ECM transport simulations. To create a clean plasma-advance front in the interstitial spaces, the secondary phase was taken as air. The ECM domain was initialized as air-filled (air volume fraction $\alpha_{\text{air}}=1$ , plasma volume fraction $\alpha_{\text{plasma}}=0$ ), and plasma displaced air over time under the inlet fields (Equation 15). The air-filled initialization of the ECM domain is adopted as a controlled numerical reference state to enable clear identification of plasma entry and displacement within the ECM region. This formulation facilitates robust front tracking and consistent quantification of plasma penetration dynamics, without aiming to reproduce the fully saturated interstitial fluid environment *in vivo*. The tumor slice geometry has three outlets; each outlet was set to a fixed pressure of 2780 Pa , following prior studies (Wu et al., 2013) and consistent with elevated interstitial pressures reported in tumors (Sven and Josipa, 2007; Stylianopoulos et al., 2018; Heldin et al., 2004). No-slip conditions are imposed on solid ECM boundaries, and surface tension effects are neglected due to the low capillary number regime considered. Material properties matched those used elsewhere in this study: plasma density and viscosity $\rho_{\text{p}}=1030\;kg/m^{3}$ and $\eta_{\text{p}}=0.001\;kg/\left(m\cdot s\right)$ (Nithiarasu, 2022); air density and viscosity $\rho_{\text{air}}=1.225\;kg/m^{3}$ and $\eta_{\text{air}}=1.8\times 10^{-5}\;kg/\left(m\cdot s\right)$ (Porterfield and Kruse, 1995). These settings, together with the inlet fields $\left\{{\bar{p}}_{\text{in}},{\bar{u}}_{\text{in}}\right\}$ and outlet pressures, define a closed initial–boundary-value problem for plasma permeation through the ECM microstructure, from which we compute the space–time evolution of plasma volume fraction, interstitial velocities, and cumulative perfusion metrics.

### 2.3 Experimental setup

**FIGURE 3:**
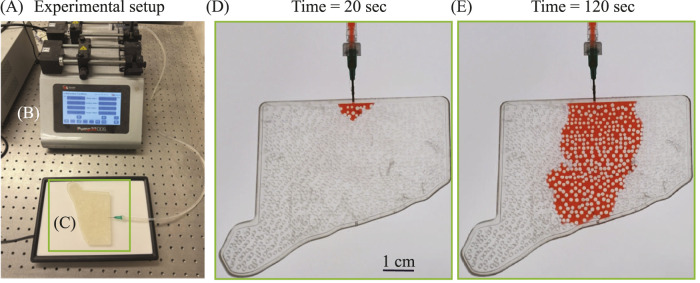
Microfluidic visualization of dye-driven perfusion through a 3D-printed tumor ECM **(A)** Experimental setup for the microfluidic test. Red–dyed deionized water was used to mimic plasma perfusion through a tumor ECM domain 3D-printed in clear photopolymer resin (C, green box). **(B)** Harvard Apparatus syringe pump delivering a constant 2 mL/min flow. **(D,E)** Time-lapse percolation within the ECM at 20 s and 120 s, respectively, showing propagation of the dyed front inside the ECM; the green frame indicates the zoomed field of view from **(C)** during flow. Scale bar 1 cm.

To replicate the physical features of a tumorous microenvironment, we fabricated a biomimetic pillar array using a Form 3+ stereolithography (SLA) resin printer (Formlabs Inc., United States). The structure (Figure 3C) was printed (3D version of Figure 2D) in clear photopolymer resin, achieving a spatial resolution of 50μ m. The design matched the packing fraction of 0.30 observed within tumors. After printing, the structure was rinsed in isopropyl alcohol and post-cured under UV light. The flow through the pillar network was driven using red-dyed deionized water as the working fluid. The fluid was supplied at a constant volumetric flow rate using a Harvard Apparatus syringe pump (Figures 3A,B), enabling precise control of perfusion conditions within the range of 1−3mL/min (Figures 3D,E are for 2mL/min ). This range ensured laminar flow within the micro-pillared structure and preserved quasi-two dimensional dynamics consistent with the assumptions of the numerical models. The transparent resin structure was illuminated from below using a diffuse white light source, while the evolving liquid front was imaged from above with a digital camera. The recorded image sequences were post-processed using image analysis software to extract the time evolution of the wetted area. Binarized frames were used to calculate the area of liquid penetration as a function of time, from which the spreading dynamics were quantified.

### 2.4 CFD-informed reverse advection-diffusion (RAD) model

In classical porous-media descriptions, such as Darcy–Starling formulations, advection is assumed to proceed in the direction of decreasing pressure from the vessel into the tissue, while diffusion proceeds down concentration gradients. However, in dense tumor extracellular matrices (ECM), elevated interstitial fluid pressure (IFP), strong geometric confinement by fiber bundles, and limited pore connectivity substantially suppress forward pressure-driven convection. As a consequence, the effective transport of plasma-borne species can oppose the direction predicted by pressure-driven models, despite the constitutive flux laws remaining unchanged (Baish et al., 2011; Sugihara-Seki and Fu, 2005). In this work, we introduce a reverse advection–diffusion (RAD) model to describe this regime. Importantly, the term “reverse” does not imply a reversal of diffusion or advection physics, nor negative diffusivity or flow inversion within the ECM. Instead, it denotes a transport regime in which the net progression of plasma-borne species within the ECM region is biased in the opposite direction to the expected forward perfusion direction predicted by pressure-driven Darcy flow, due to geometric obstruction and elevated interstitial resistance. The RAD model is therefore a conceptual and mathematical framework for capturing transport opposition relative to classical perfusion assumptions, rather than a direct solver for interstitial biofluid flow. The RAD model is formulated as a reduced-order theoretical transport surrogate, distinct from the multiphase ECM simulations described in Section 2.2.2. No momentum or pressure equations are solved in the ECM domain. Instead, solute transport is modeled directly through an advection–diffusion equation with prescribed advection strength, allowing isolation of transport kinetics without resolving multiphase or interstitial flow dynamics. In this sense, the RAD model is not a multiphase ECM model and is not dynamically coupled to the plasma–air displacement simulations of Section 2.2.2. Rather, it provides a simplified representation of plasma progression once entry into the ECM has occurred. The governing transport equation for solute concentration c(x,y,t) is

$$\frac{\partial c\left(x,y,t\right)}{\partial t}+\omega_{x}\left(x,y\right)\;\frac{\partial c}{\partial x}+\omega_{y}\left(x,y\right)\;\frac{\partial c}{\partial y}-D\;\left(\frac{\partial^{2}c}{\partial x^{2}}+\frac{\partial^{2}c}{\partial y^{2}}\right)=0,$$ (16)

where D is the effective diffusivity, and $\omega \left(x,y\right)=\left(\omega_{x},\omega_{y}\right)$ is the imposed advection field; in practice we take $\omega_{x}=0$ and advection is imposed solely in the direction normal to the fenestra through $\omega_{y}$ . This imposed advection does not represent resolved convection within the ECM bulk; rather, it serves as a phenomenological representation of the transport-driving influence of plasma entry at the vessel–ECM interface. The RAD model is termed “CFD-informed” as the magnitude of $\omega_{y}$ is derived directly from vessel-scale multiphase CFD simulations. Specifically, fifteen independent tumor-vessel CFD simulations are performed with varying fenestra locations. From each simulation, the magnitude of the plasma entry velocity at the fenestra is extracted. A single characteristic advection scale is then defined as the arithmetic ensemble average of these velocity magnitudes across all vessel geometries, and this value is used to prescribe $\omega_{y}$ in Equation 16. No temporal averaging is performed, and no spatial velocity field is transferred into the ECM domain. Thus, CFD-informed here denotes parameterization of transport strength using CFD-derived entry conditions, not volumetric or transient coupling between CFD and ECM models. The RAD model is posed on a reduced-order rectangular ECM domain of size $10\times 5\;\mu m^{2}$ with a 0.3 μm fenestra through which plasma enters. A total of 40 circular fiber inclusions are placed with minimum spacing (no overlap) to maintain a packing fraction ϕ=0.30 (Andersson et al., 2008; Nier et al., 2016; Spatarelu et al., 2019; Yeasin, 2025), yielding an average fiber radius $\bar{r}\approx 0.345\;\mu m$ . The geometry (Figure 8A) is reconstructed in Wolfram Mathematica and meshed (Figures 8 B,C) via ToElementMesh into an unstructured triangular grid ( $∼5.5\times 10^{4}$ elements), providing sufficient resolution near the fenestra and fiber boundaries. We initialize with c(x,y,0)=0 and apply a constant inlet concentration of $8.0\times 10^{13}$ —twelve orders of magnitude above the physiological protein concentration in plasma ( ∼80 mg/mL (Leeman et al., 2018))–at the fenestra. To match CFD timescales in this reduced domain, A constant inlet concentration is prescribed at the fenestra, while zero-flux (Neumann) conditions are enforced on all fiber surfaces and lateral boundaries to prevent artificial back-diffusion. The RAD model is designed to reproduce the spatiotemporal progression trends of plasma transport observed in CFD and microfluidic experiments, rather than absolute concentration levels. Accordingly, inlet concentration magnitude and diffusivity ( $D=1270\;\mu m^{2}/s$ (Muraoka et al., 2008)) are treated as effective parameters calibrated to match CFD-resolved transport timescales.

Finally, to verify consistency between the theoretical RAD framework and full numerical transport simulations under identical boundary forcing, a two-phase Eulerian CFD simulation was performed on the same reduced geometry using ANSYS Inc, 2023 R1. The domain was constructed in ANSYS Inc, 2023 R1 and discretized with an unstructured triangular mesh (approximately $7.1\times 10^{4}$ elements) with local refinement near the fenestra and along the patched-wall segments. To accommodate the concentration inlet prescribed by the theoretical model, only the outlet boundary condition was modified for this validation case; all other settings (solver options, material properties, numerics) were kept identical to Section 2.2.2. Geometry and boundary placements were identical to those used for the RAD domain. This validation exercise does not constitute coupling between models, but rather demonstrates that the simplified RAD formulation reproduces key transport trends when driven by the same fenestral entry conditions.

## 3 Results

### 3.1 Comparison of plasma perfusion from vessel to ECM with and without EHD

**FIGURE 4:**
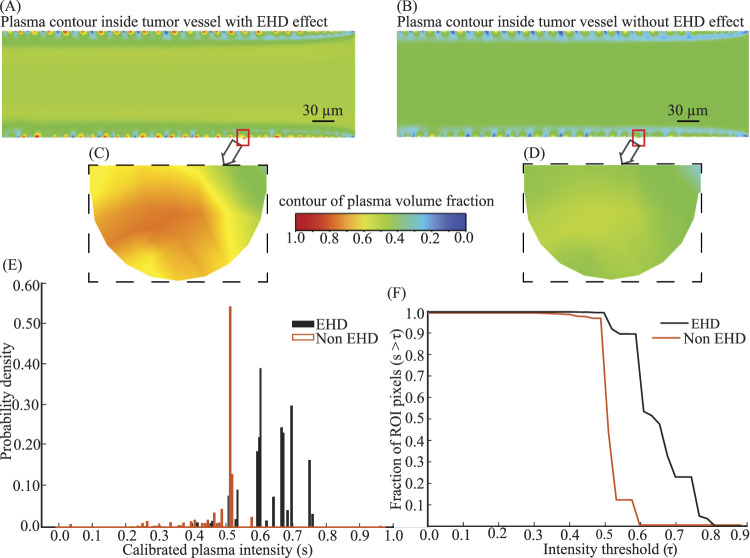
EHD flow yields enhanced, more persistent plasma penetration at the fenestra compared with non-EHD flow **(A,B)** Plasma (primary phase) contour inside the tumor vessel with EHD **(A)** and without EHD **(B)**. **(C,D)** Zoomed fenestra ROI from panels A and B (same simulation Model 5). The dashed rectangle delineates the identical physical area used for pixel counting and threshold analysis. **(E)** Fenestra-ROI intensity distributions show a clear rightward shift under EHD with minimal overlap, indicating stronger local plasma penetration; mean intensities 0.650 (EHD) and 0.525 (non-EHD). **(F)** Threshold (survival) curves summarize robustness across cutoffs the EHD curve dominates across thresholds, and the area-between-curves (ABC) equals 0.125 (Equations 21-22), demonstrating more persistent high-intensity pixels only with EHD.

We quantify how the electrohydrodynamic (EHD) force in the tumor vessel modifies the plasma delivered through the fenestra into the ECM, relative to an otherwise identical non-EHD baseline. Using the representative geometry (Model 5), we select the same region of interest (ROI)–the fenestra–in both simulations (Figures 4A,B). Pixel colors in the ROI are mapped to a dimensionless plasma intensity S∈[0,1] producing two paired empirical distributions over the identical ROI: $S_{\text{EHD}}$ and $S_{\text{non}-\text{EHD}}$ . The histogram comparison in Figure 4E shows a decisive rightward shift with EHD. Denote the sample means by ${\bar{S}}_{\text{EHD}}$ and ${\bar{S}}_{\text{non}-\text{EHD}}$ , and standard deviations by $s_{\text{EHD}}$ and $s_{\text{non}-\text{EHD}}$ and let $s_{p}$ be the pooled standard deviation. The standardized mean difference (Cohen’s d ) (Cohen, 2013) is

$$d=\frac{{\bar{S}}_{\text{EHD}}-{\bar{S}}_{\text{non}-\text{EHD}}}{s_{p}},\;s_{p}=\sqrt{\frac{\left(n_{\text{EHD}}-1\right)s_{\text{EHD}}^{2}+\left(n_{\text{non}-\text{ EHD}}-1\right)s_{\text{non}-\text{ EHD}}^{2}}{\;n_{\text{EHD}}+n_{\text{non}-\text{EHD}}-2\;}}.$$ (17)

For Model 5, $\bar{S}$ increases from 0.525 (non–EHD) to 0.650 (EHD), yielding d=2.05 using Equation 17 (“very large” separation). A threshold-free dominance metric is the probability of superiority (AUC),

$$AUC=\mathrm{Pr}\;\left(S_{\text{EHD}}>S_{\text{non}-\text{EHD}}\right),$$ (18)

from which Cliff’s delta follows as

δ=2 AUC−1. (19)

Here, AUC=0.902 (from Equation 18) and δ=0.804 (from Equation 19) indicate minimal overlap between conditions. A robust location shift is summarized by the Hodges–Lehmann median (Equation 20) difference,

$$\Delta_{\text{HL}}=\text{median }\;\left\{\;S^{\text{EHD}}-S^{\text{non}-\text{EHD}}\;\right\}=+0.107.$$ (20)

To assess areal coverage at clinically relevant intensities, we examine the exceedance (survival) curves (Figure 4F) over a threshold τ∈[0,1] :

$$E_{•}\left(\tau \right)=\frac{1}{N_{\text{pix}}}\;\#\left\{\;x\in ROI:\;S^{\left(•\right)}\left(x\right)>\tau \;\right\},\;\left(•\right)\in \left\{EHD,non-EHD\right\}.$$ (21)

Figure 4F shows $E_{\text{EHD}}\left(\tau \right)$ (black) lies strictly above $E_{\text{non}}\left(\tau \right)$ (orange) across the inspected range, i.e., larger high-intensity area for any chosen cutoff. The area between curves (ABC) provides a cutoff-agnostic summary:

$$ABC=\int_{0}^{1}\;\left[E_{\text{EHD}}\left(\tau \right)-E_{\text{non}}\left(\tau \right)\right]\;d\tau =0.125.$$ (22)

At a stringent threshold of τ=0.7 , the high-intensity fraction is 22.92% (EHD) versus 0.0% (non-EHD), clearly showing a strongly perfused area under EHD. In Figure 4, panels E–F jointly indicate that EHD (via patched, glycocalyx-mediated wall forcing) increases both how much of the fenestra area attains high plasma fraction (larger exceedance across τ ) and how strongly it is filled (right-shifted intensity distribution). These inlet-side gains are exactly the mechanism that yields the faster and broader percolation observed downstream in the ECM domain (Section 3.2).

**FIGURE 5:**
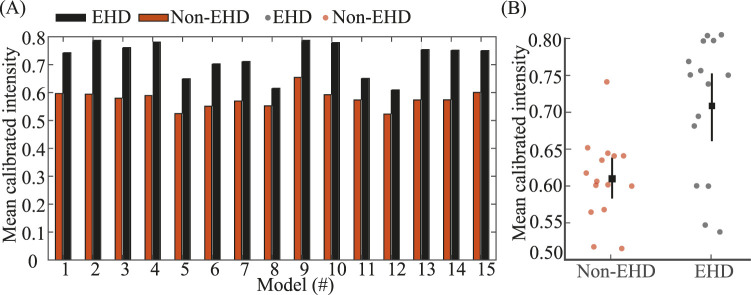
Structural sensitivity of EHD-induced plasma extravasation across vessel geometries. EHD flow consistently yields higher mean plasma penetration than non-EHD across all 15 models: **(A)** For models 1–15 (fenestra center translated from near inlet to near outlet), grouped bars compare Non–EHD and EHD mean calibrated intensity for each model; higher values indicate greater plasma penetration. EHD exceeds Non–EHD in every model. **(B)** Per–model means (dots) with grand mean and 95% bootstrap CI (square with whiskers) for each condition. Across models, mean Non–EHD =0.576 and mean EHD =0.722 (mean Δ=0.146 , 95% CI [0.124, 0.166] ; Wilcoxon signed–rank $p=6.1\times 10^{-5}$ ).

*Structural sensitivity:* across all 15 models, Figures 5A,B demonstrates that the mean calibrated intensity under EHD is consistently higher than under Non–EHD. Figure 5A shows this pattern model–by–model, while panel B summarizes the across–model effect: grand means of 0.722 (EHD) versus 0.576 (Non–EHD), with a mean improvement Δ=0.146 (95% CI [0.124, 0.166] ; Wilcoxon signed–rank $p=6.1\times 10^{-5}$ ). Taken together, these results indicate that the EHD-induced enhancement of plasma extravasation is robust to changes in fenestra location and vessel geometry, rather than being specific to a single configuration. Our objective here is to establish the condition effect (EHD vs. Non–EHD), not to optimize fenestra placement; accordingly, we do not rank or compare the EHD models against one another by location.

### 3.2 Plasma percolation inside the tumor ECM domain

**FIGURE 6:**
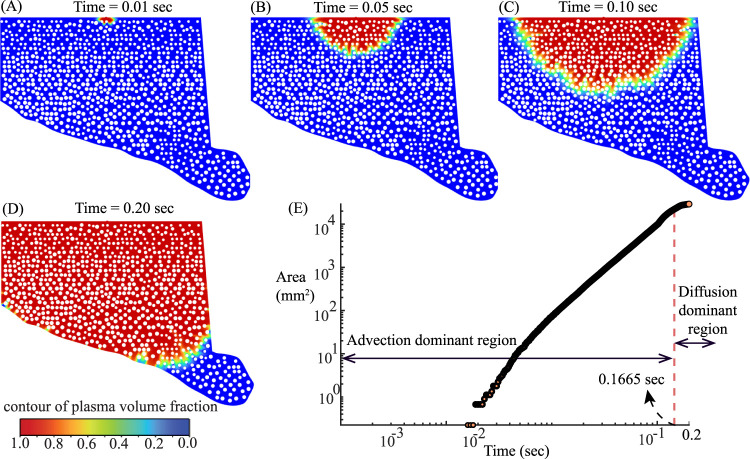
Spatiotemporal spreading of plasma in the tumor ECM shows an advection-to-diffusion transition in wetted area growth: **(A–D)** Contours of plasma volume fraction at **(A)** 0.01 s, **(B)** 0.05 s, **(C)** 0.10 s, and **(D)** 0.20 s. White circles denote impermeable fibers; blue is unfilled interstitium. The 0–1 color bar maps local plasma fraction. The wetted region expands from a thin inlet plume to a broad front, illustrating a qualitative shift from advection-driven advance toward diffusion-controlled spreading. **(E)** Wetted area versus time on log–log axes. The red dashed line marks the transition time $t^{*}=1.665\times 10^{-1}\;sec$ , separating the advection-dominant region (left) from the diffusion dominant region (right).

With the tumor-domain inlet fields $\left({\bar{p}}_{\text{in}}\left(t_{n}\right),\;{\bar{u}}_{\text{in}}\left(t_{n}\right)\right)$ constructed from the N=15 vessel simulations (Section 2.2.2; Equation 15), the flow advances the air–plasma interface through the tumor ECM domain. The spatiotemporal evolution exhibits a coherent percolation pattern: an early thin, inlet-aligned jet penetrates along preferential low-resistance pathways; intermediate times show lateral broadening and progressive saturation of interstitial voids; and late times approach a nearly uniform, high–plasma-fraction interior behind the front. This progression is visualized in Figures 6A–D via contours of local plasma volume fraction, where red denotes the advancing high-fraction front, yellow/green the graded rim, and blue the not-yet-perfused interstitium. To quantify front advance and rim thickening, we compute an image-based color occupancy ratio for each frame by normalizing the blue (unfilled) pixels to B=1 and reporting the triplet R:(G+Y):B after excluding white pores from the counts. A rise in R:B signals deeper filling, and a rise in (G+Y):B signals a thickening front. As summarized in Table 2, R:B grows monotonically from $\approx 2\times 10^{-3}$ at t=0.01 sec to ≈6.83 at t=0.20 sec, with a concurrent increase in (G+Y):B . Consistent with the visual maps, early times are advection-dominant (small R:B , thin inlet-aligned plume); by mid-times the rim thickens as cross-stream spreading increases; at late time most accessible voids are filled (blue nearly exhausted) and the field behind the front approaches homogeneous red.

**TABLE 2:** Color-occupancy ratios for Figures 6A–D. Ratios are computed from pixel counts with blue normalized to 1; white pore pixels are excluded. Increasing R:B and (G+Y):B indicate deeper penetration and a thickening front.

Panel	Time (sec)	R:B	(G+Y):B	R:(G+Y):B
(A)	0.01	0.0020	0.0006	0.0020:0.0006:1
(B)	0.05	0.0740	0.0096	0.0740:0.0096:1
(C)	0.10	0.4386	0.0396	0.4386:0.0396:1
(D)	0.20	6.8276	0.0930	6.8276:0.0930:1

A complementary, domain–integrated metric is the wetted area starting with A(t) (Figure 6E) of non-blue pixels. On log–log axes we analyze $\left(x_{i},y_{i}\right)=\left(\log_{10}t_{i},\log_{10}A_{i}\right)$ so that a power law $A∼t^{\alpha }$ appears as a straight line:

$$y=m\;x+b\;⟺\;A\left(t\right)=10^{\;b}\;t^{\;m},$$ (23)

with exponent α=m . A two–segment least–squares fit in (x,y) identifies a break point $t^{*}$ that minimizes the total residual sum of squares. Using this procedure we obtain $t^{*}=0.1665\;sec$ , which partitions the record into advection region: t $\in \left[1.60\times 10^{-3},\;1.664\times 10^{-1}\right]$ sec, and diffusion dominant region: t $\in \left[1.666\times 10^{-1},\;2.00\times 10^{-1}\right]$ sec. Within any ${\left\{\left(x_{i},y_{i}\right)\right\}}_{i=1}^{n}$ , ordinary least squares gives (see Table 3)

$$\bar{x}=\frac{1}{n}\underset{i}{\sum }x_{i},\;\bar{y}=\frac{1}{n}\underset{i}{\sum }y_{i},\;m=\frac{\sum_{i}\left(x_{i}-\bar{x}\right)\left(y_{i}-\bar{y}\right)}{\sum_{i}{\left(x_{i}-\bar{x}\right)}^{2}},\;b=\bar{y}-m\;\bar{x}.$$ (24)

**TABLE 3:** Segment-wise fits on $\left(\log_{10}t,\log_{10}A\right)$ for $\log_{10}A=m\;\log_{10}t+b$ .

Segment	t –range (sec)	m	b	$R^{2}$
Advection region	$\left[1.60\times 10^{-3},\;1.664\times 10^{-1}\right]$	2.21	5.23	0.99
Diffusion dominant region	$\left[1.666\times 10^{-1},\;2.00\times 10^{-1}\right]$	0.41	3.75	0.98

To summarize the transition at $t^{*}$ , we report the one–sided model predictions, $A_{\text{adv}}\left(t^{*}\right)=10^{\;b_{\text{adv}}}{\left(t^{*}\right)}^{m_{\text{adv}}}\approx 3.23\times 10^{3}\;{mm}^{2},A_{\text{diff}}\left(t^{*}\right)=10^{\;b_{\text{diff}}}{\left(t^{*}\right)}^{m_{\text{diff}}}\approx 2.70\times 10^{3}\;{mm}^{2}$ . The advection side extrapolation is therefore higher at the breakpoint (offset ≈20% relative to the diffusion-side value; ≈18% relative to the mean of the two), which is typical for an unconstrained two–line fit and indicates an abrupt change in kinetics rather than a perfectly continuous crossover. The effective exponent drops from super–linear to subunit from Equations 23-24 ( m:2.21 to 0.41), consistent with a shift from advection–driven growth to a diffusion–controlled late regime; this interpretation agrees with the RGB composition (persistent graded rim).

### 3.3 Experiment–CFD validation of wetted-area growth

**FIGURE 7:**
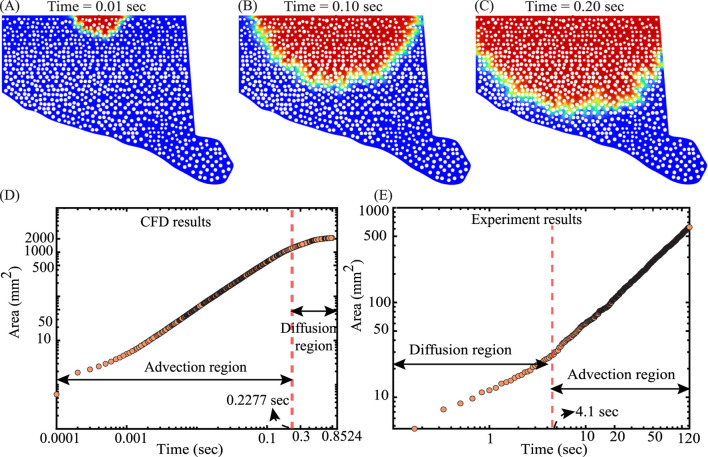
CFD and experiments both show advection-dominated growth regimes in ECM wetting, with different transition times: **(A–C)** Snapshots show front advance at t=0.01, 0.10, 0.20,sec respectively. **(D)** log–log growth of area A(t) with an advection-dominated regime transitioning to diffusion at $t_{\text{CFD}}^{*}=0.2277\;sec$ . **(E)** Early diffusion-dominated growth $\left(A∼t^{1/2}\right)$ transitioning to advection (A∼t) at $t_{\text{exp}}^{*}=4.1\;sec$ .

Top view images (Figures 3D,E) from the experiments were segmented to obtain wetted pixel counts over time and converted to physical area, yielding A(t) in Figure 7E. The experiment exhibits advection dominated growth overall, with an early shoulder consistent with $A\left(t\right)∼t^{1/2}$ before a clear linear trend A(t)∼t becomes established; we identify the knee at $t_{\text{exp}}^{*}=4.1\;sec$ . For a like-for-like benchmark, we ran a separate 2D CFD configuration (distinct from Section 2.2.2) using a pressure-driven inlet to match the experimental 2 mL/min ; plasma and air properties were unchanged, and the front evolution appears in Figures 7A–C. Applying the same area-extraction to the simulation yields the same qualitative behavior; advection-dominated growth with a brief early shoulder—and a much earlier knee at $t_{\text{CFD}}^{*}=0.2277\;sec$ (Figure 7D). Thus, both experiment and CFD are advection-dominated; the primary discrepancy is the transition timescale ( $t_{\text{exp}}^{*}$ versus $t_{\text{CFD}}^{*}$ ), which we analyze in Section 4.4 in terms of inlet start-up/wetting effects and geometric idealization.

### 3.4 Comparison of theoretical model predictions with corresponding numerical simulation outcomes

**FIGURE 8:**
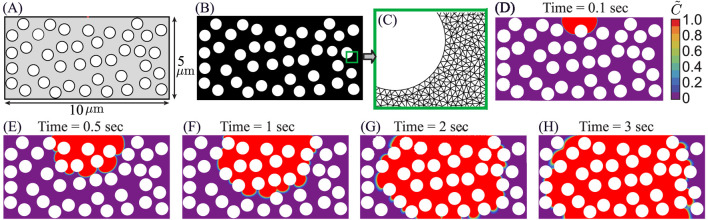
CFD-informed reverse advection-diffusion RAD model showing solute plume evolution through a reduced-order fiber-dense tumor ECM: **(A)** The simplified rectangular computational domain $\left(10\times 5\;\mu m^{2}\right)$ used for the reduced-order RAD model, containing randomly distributed circular ECM fiber bundles occupying 30% of the rectangular domain area. All fibers have equal radius and act as impermeable structures, mimicking dense tumor stroma and restricting solute motion. **(B)** The high-resolution unstructured triangular finite-element mesh generated for the theoretical analysis. Whereas **(C)** provides a zoom view of the mesh in the fenestra region, highlighting refined discretization around fiber boundaries to resolve steep concentration gradients. **(D–H)** The predicted spatiotemporal evolution of plasma-borne solute under the advection-diffusion transport model at t=0.1 sec, 0.5 sec, 1.0 sec, 2.0 sec, and 3.0 sec , respectively, where $\tilde{C}$ represent the normalized concentration field.

**FIGURE 9:**
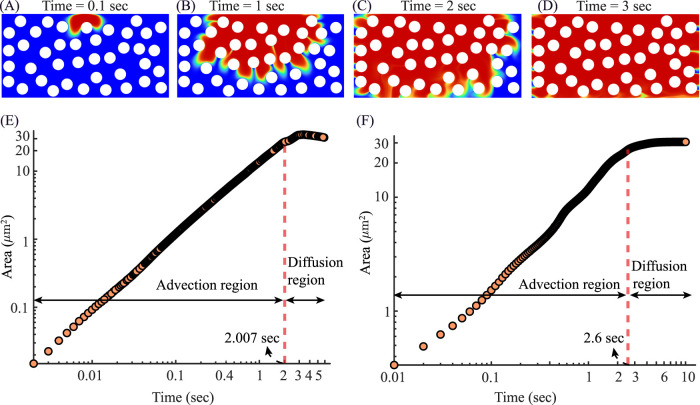
Comparison of CFD and RAD model for plasma transport transition: **(A–D)** show the CFD-predicted plasma propagation at t=0.1,1,2 , and 3 sec , respectively. Plasma first penetrates swiftly along preferential pathways between fibers, then progressively spreads laterally and fills the matrix, reflecting a transition from advection-dominated entry to diffusion-driven equilibration. **(E)** Shows the occupied interstitial area A(t) as a function of time for the CFD solution, demonstrating a steep early-time growth regime followed by a slowed diffusion-controlled regime as the domain fills. **(F)** Shows the corresponding theoretical RAD model prediction for A(t) , showing the same trend as the CFD solution.

The reverse advection–diffusion (RAD) model reproduces the same two-stage transport seen in the like-for-like CFD of the simplified rectangular domain: an inlet-aligned, advection-driven entry plume followed by diffusion-dominated homogenization as the interior fills (theory snapshots in Figures 8D–H; CFD snapshots in Figures 9A–D). To quantify kinetics, we track the occupied interstitial area A(t) for both models on log–log axes (Figures 9E,F). In each case the slope transitions from a steep, advection-driven regime to a shallow, diffusion-controlled regime, with breakpoints at $t_{\text{CFD}}^{*}\approx 2.007\;sec$ (Figure 9E) and $t_{\text{th}}^{*}\approx 2.60\;sec$ (Figure 9F).

We summarize the post-transition timescale offset by the simple ratio

$$k=\frac{t_{\text{th}}^{*}}{t_{\text{CFD}}^{*}}=\frac{2.60}{2.007}\approx 1.295,$$ (25)

indicating that the theoretical curve evolves about 1.295 times more slowly than the CFD result on this domain. If we rescale the theoretical time axis by this factor,

$$t_{\text{th,scaled}}=\frac{t_{\text{th}}}{k},$$ (26)

it will bring the A(t) trajectories into close agreement in both the early advection regime and the late diffusion-dominated regime (Equations 25–26). Thus, the reduced-order RAD model captures the same mechanistic shift observed in CFD and, after a single temporal calibration, serves as an accurate and computationally efficient surrogate for fully resolved flow simulations on this geometry.

## 4 Discussion

### 4.1 Diffusion dominant late regime with subunit area scaling

In the late regime ( $t>t^{*}=0.1665$ sec)), wetted–area growth is governed by diffusion even though the log–log exponent is subunit $\left(m_{\text{diff}}\approx 0.41\right)$ . A persistent graded rim (green + yellow) leading the bulk (red), quantified by slowly varying color ratios (Table 4), is consistent with diffusion–limited penetration through a tortuous, fiber–dense matrix. In diffusion–limited flow, the characteristic penetration length obeys $\ell \left(t\right)∼\sqrt{D\;t}$ , so observables that scale with length (e.g., a band–like front across a roughly fixed width) grow as $t^{1/2}$ , not linearly (Crank, 1979). Consequently, the area can grow sublinearly (m<1) and still be diffusion–controlled. In ideal band–like geometries one expects $A\propto t^{1/2}$ (m=0.5) ; values below 0.5 are expected in complex matrices where tortuosity, dead–ends, or partial channelization slow the effective front (subdiffusion) (Sahimi, 2011; Metzler and Klafter, 2000; Berkowitz et al., 2000; Edery et al., 2014).

**TABLE 4:** Color–composition ratios in the diffusion–dominant region in Figure 10.

Panel	Time (sec)	R:B	(G+Y):B	(G+Y):R
A	0.1666	4.63	0.161	0.0348
B	0.1800	5.63	0.147	0.0261
C	0.1900	6.188	0.137	0.0221
D	0.2000	6.75	0.130	0.0193

**FIGURE 10:**
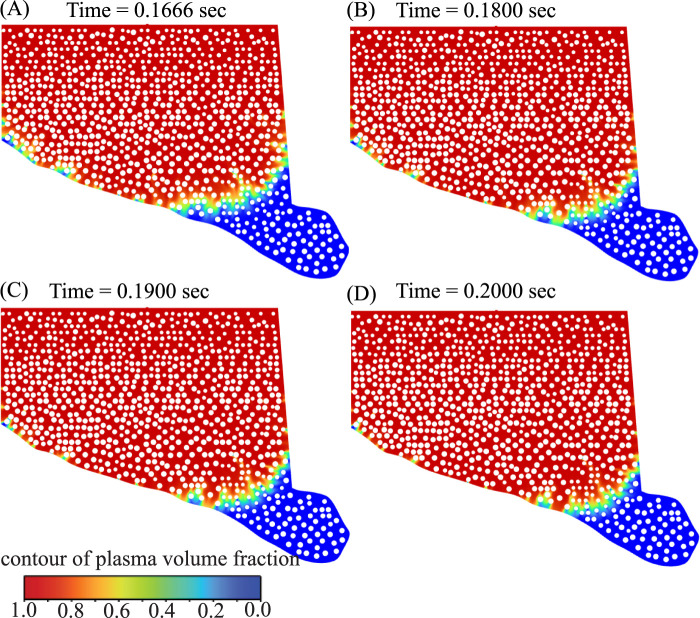
Temporal evolution of plasma penetration inside tumor ECM domain at different flow times: Panels show snapshots of plasma perfusion inside the tumor domain at **(A)** t = 0.1666 s, **(B)** 0.1800 s, **(C)** 0.1900 s, and **(D)** 0.2000 s within the diffusion-dominated region. Red denotes the high-fraction bulk, green + yellow denote the graded rim, and blue denotes the unperfused region. Panels show a persistent graded rim leading a slowly advancing bulk with a gradual recession of blue—indicates gradient-limited (diffusive) penetration through a tortuous, fiber dense matrix, consistent with subunit area-growth scaling.

Independent image–based composition metrics corroborate a diffusion–controlled tail. Post $t^{*}$ snapshots show a persistent graded rim (green + yellow) leading the high–fraction bulk (red) while the unperfused region (blue) depletes gradually. Quantitatively, the R:B increases only slowly from 4.63 to 6.75 between t = 0.1666 and 0.2000 sec, and the (G+Y):B remains appreciable (0.161–0.130). Crucially, the rim–to–bulk ratio ((G+Y):R) stays non-vanishing and declines only mildly ∼3.5%→1.9% , indicating a broad, gradient–limited front rather than a sharp advective discontinuity at high Péclet number.

### 4.2 Modeling assumptions for blood rheology

In the present framework, red and white blood cells are represented as dispersed phases to capture phase fraction distributions, and electrohydrodynamic (EHD) effects near fenestrae. However, the microscale mechanisms responsible for blood’s non-Newtonian behavior—such as RBC deformation, aggregation, and membrane dynamics—are not explicitly resolved. Accordingly, the Carreau–Yasuda viscosity law is introduced phenomenologically as a constitutive closure to represent the net rheological response observed experimentally, rather than as a property emerging self-consistently from the multiphase dynamics. This separation of transport representation and constitutive rheology avoids double-counting and is consistent with established continuum blood flow modeling practices.

### 4.3 Modeling assumptions and interpretation of plasma transport inside ECM region

Within the current framework, plasma transport within the tumor extracellular matrix (ECM) is modeled using a two-phase transport formulation to provide a well-defined reference state for quantifying plasma entry and penetration from the vessel wall. Although the *in vivo* ECM is continuously saturated with interstitial fluid, the air phase used here should be interpreted as a numerical placeholder representing the resident interstitial volume rather than a physical gas phase. In this sense, the model captures liquid–liquid replacement behavior, with plasma displacing an initially present fluid, but does so through an immiscible front-tracking formulation rather than a concentration-based mixing model. This approach enables clear identification of transport initiation and penetration depth, avoids numerical ambiguity associated with weak concentration gradients, and facilitates consistent comparison across vessel realizations and inlet conditions. Consequently, the resulting plasma volume fraction and penetration metrics should be interpreted as relative indicators of ECM perfusion efficiency and transport trends, and are generalizable in a comparative sense rather than as direct predictions of absolute *in vivo* interstitial solute concentrations. Future extensions of the framework may incorporate fully saturated liquid–liquid mixing formulations to recover detailed concentration fields while retaining the multiscale vessel–ECM coupling strategy developed in this work.

### 4.4 On the experiment–CFD timescale discrepancy and thickness scaling

We first quantify the temporal discrepancy by comparing the post-transition analysis. The corresponding timescale ratio is

$$\frac{\Delta t_{\text{experiment}}}{\Delta t_{\text{CFD}}}=\frac{120-t_{\text{exp}}^{*}}{t_{\text{CFD}}^{*}-0}=\frac{120-4.1}{0.2277-0}=\frac{115.9}{0.2277}\approx 5.09\times 10^{2}.$$ (27)

This result indicates that the experimental timescale is approximately 509 times (from Equation 27) longer than the computational timescale, emphasizing the profound effect of physical constraints present in the 3D experimental setup but absent in the 2D numerical model.

Under the constant–flow (pump–driven) conditions of the experiment, mass conservation in a thin layer of thickness H gives $Q=\varepsilon H\;\frac{dA}{dt}$ , so the characteristic time obeys Δt∝H at leading order. Interpreting the 2D CFD as a per–depth surrogate of effective thickness $h_{\text{eff}}$ , the transition–time ratio therefore scales as

$$\frac{t_{\text{exp}}^{*}}{t_{\text{CFD}}^{*}}\approx \frac{H_{\text{exp}}}{h_{\text{eff}}}.$$ (28)

**FIGURE 11:**
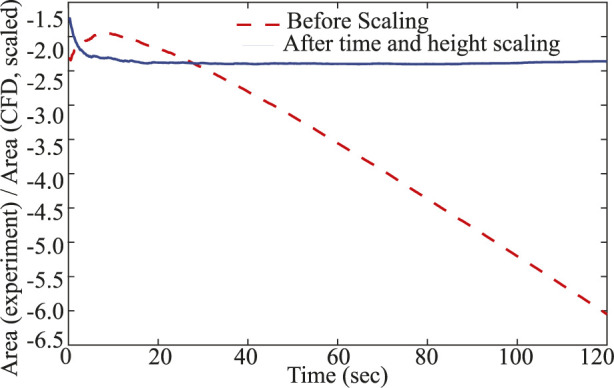
Scaling reconciliation of wetted-area growth between experiment and CFD: Wetted-area dynamics align between experiment and CFD after time and thickness scaling. Red dashed: experiment vs. raw CFD (before scaling). Blue solid: experiment vs. scaled CFD (after scaling). Post-scaling, the offset is nearly constant and the trends coincide, indicating matched advection-dominated growth.

With $H_{\text{experiment}}=7.5\;mm$ = 7500 μm and the measured ratio $\approx 1.85\times 10^{2}$ , we infer from Equation 28

$$h_{\text{eff}}=\frac{H_{\text{exp}}}{\Delta t_{\text{exp}}/\Delta t_{\text{CFD}}}=\frac{7.5\;mm}{5.09\times 10^{2}}\approx 15\;\mu m,$$ (29)

which explains the $O\left(10^{2}\right)$ gap directly from the 3D–to–2D thickness difference. To reconcile the curves graphically (Figure 11), we rescale CFD time and area (from Equations 28–29) by

$$f_{t}=\frac{\Delta t_{\text{exp}}}{\Delta t_{\text{CFD}}}\approx 5.09\times 10^{2},\;t_{\text{CFD,scaled}}=t_{\text{CFD}}\;f_{t},$$ (30)

$$f_{a}=\frac{H_{\text{exp}}}{h_{\text{eff}}}\approx 5\times 10^{2},\;A_{\text{CFD,scaled}}=A_{\text{CFD}}\;f_{a},$$ (31)

and plot the residual as (using Equations 30–31)

$$\log_{10}\;\left(A_{\text{exp}}/A_{\text{CFD,scaled}}\right)=\log_{10}A_{\text{exp}}-\left(\log_{10}A_{\text{CFD}}+\log_{10}f_{a}\right).$$

After an initial transient, the residual flattens (blue curve), indicating consistent advection–dominated growth once thickness and time are reconciled; any remaining offset is attributable to early–time device effects (pump start–up/compliance, wetting/pinning and capillary entry on the resin surface, transient air evacuation) that are absent from the idealized 2D model.

### 4.5 On the use of a large inlet concentration in the RAD model

The reverse advection-diffusion (RAD) model matches CFD on both the advective and diffusion regimes, indicating that key transport physics are retained without full Navier–Stokes resolution; hence RAD is well-suited for rapid sweeps and design use. To theoretically model the diffusive component for the perfusion dynamics within the extracellular domain, we have imposed a finitely large value ( $c_{\text{in}}=8.0\times 10^{13}$ in model units) for plasma concentration at time t=0 as a pragmatic approximation (Crank, 1979) mimicking a persistent, high concentration fenestral ‘point’ source. A sufficiently large (but finite) value for initial inlet concentration creates a steep concentration gradient that effectively drives diffusion while maintaining the stability and robustness of the analytical solver. This approach aligns with standard practice in transport modeling, where high boundary concentrations are employed as Dirichlet boundary conditions to represent sustained sources, thus allowing for a realistic yet computationally feasible launch point for the complex intra-tumoral transport process without sacrificing physical relevance.

The present surrogate intentionally simplifies geometry and driving fields to isolate advection–diffusion obstruction physics: an idealized rectangle with uniformly sized, non-contacting circular fibers and a spatially simple inlet advection profile. Future work will incorporate histology-derived fiber statistics (size distribution, alignment, and contacts) and spatially varying ω(x,y) , informed by localized CFD or microfluidic measurements, thereby extending RAD from rapid screening toward tissue-specific predictions while preserving computational tractability.

### 4.6 Specimen-specific tumor vessel-ECM geometry reconstruction: future model extensions

**FIGURE 12:**
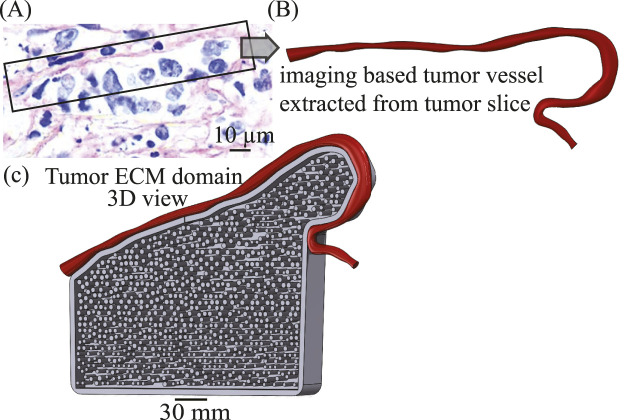
Histology-to-geometry reconstruction pipeline for future specimen-specific vessel–ECM modeling. **(A)** H&E histology of an orthotopic human pancreatic tumor slice illustrating irregular microvasculature; this specimen informed the vessel geometry. **(B)** Imaging-based vessel extracted from the tumor slice and used to define the curved vessel–ECM interface. **(C)** Tumor ECM domain (3D view) populated with circular fiber bundles to achieve the prescribed packing fraction; the curved red vessel indicates the histology-informed vessel–ECM boundary used to locate the fenestra.

While the present study employs idealized vessel–ECM geometries to enable controlled mechanistic interrogation of plasma transport and electrohydrodynamic effects, we have also developed a histology-to-geometry reconstruction pipeline intended for future specimen-specific modeling. Representative H&E-stained tumor sections (Figures 1B, 2C) were obtained from pancreatic tumor tissue, which was collected, fixed in formaldehyde for 24 h, paraffin embedded, and sectioned at 5 μ m. Sections were deparaffinized in Histo® Clear and ethanol, rehydrated, stained with hematoxylin (5 min) and eosin (1 min), dehydrated in 95%–100% ethanol, cleared in xylene, mounted, and imaged using a Zeiss Axio Observer Z1 microscope, following the same histological preparation and imaging protocol described in our prior work (Akash et al., 2023). An in-house image-analysis framework was applied to these histological sections to detect the luminal periphery using intensity-based edge detection combined with morphological cleanup. The extracted edge contours were converted into ordered coordinate sets along the vessel wall and imported into a CAD environment, where they were aligned to a common reference frame. A spline-based interpolation procedure was then used to generate a continuous vessel-wall representation with $C^{1}$ continuity, reducing pixelation artifacts inherent to raster-based images while preserving geometric fidelity. This pipeline enables reconstruction of curved vessel–ECM interfaces directly from histological data (Figures 12A,B) and supports the generation of adjacent ECM domains populated with fiber bundles at prescribed packing fractions (Figure 12C). Importantly, this histology-informed reconstruction capability is presented here as a methodological extension and is not utilized in the numerical simulations reported in the present study. The simulations intentionally rely on idealized geometries to isolate electrohydrodynamic and multiphase transport mechanisms, thereby avoiding confounding anatomical variability. Integration of specimen-specific vessel morphologies derived from this pipeline into the multiscale transport framework represents a natural and ongoing direction for future investigation.

### 4.7 Scope of the study: fluid-mechanical foundations vs. pharmacological modeling

It is important to clarify the scope and interpretation of the present study. This work does not aim to predict drug delivery or molecular pharmacokinetics within tumor tissue. Instead, it focuses on the fundamental fluid-mechanical mechanisms governing plasma perfusion and transport within the tumor extracellular matrix (ECM). Plasma is treated as the carrier phase whose ingress, penetration, and redistribution establish the baseline transport capacity of the tumor extracellular matrix (ECM), independent of any specific solute payload. Accordingly, plasma volume fraction, penetration depth, and front advancement are interpreted as mechanistic indicators of ECM perfusion efficiency rather than as direct surrogates for drug concentration or therapeutic efficacy. By isolating plasma transport physics, the framework enables systematic interrogation of how vessel-scale flow conditions and localized electrohydrodynamic effects bias interstitial transport pathways, providing mechanistic insight that can later inform, but does not itself constitute, drug-delivery modeling.

## 5 The main takeaways

We integrate three-phase, glycocalyx-patched (EHD) CFD with a histology-informed ECM domain to quantify how endothelial EHD alters plasma delivery at a fenestra and its downstream percolation. We then use a CFD-informed reverse advection–diffusion (RAD) model as a corroborative, geometry-aware surrogate for rapid exploration. The key findings are that across all 15 fenestra positions, adding EHD increases the inlet-side mean calibrated plasma intensity from 0.576 (Non-EHD) to 0.722 (EHD), representing a 25.34% gain. Downstream, both the ECM simulations and a microfluidic benchmark exhibit the same two-stage transport (advection-dominated entry followed by diffusion-driven spread), and the CFD-informed RAD reproduces these kinetics successfully.

In future work, we will extend the ECM-domain simulations to fully three-dimensional geometries, capturing transverse leakage paths and thickness-dependent effects that are absent in 2D. The reverse advection–diffusion model will be augmented with explicit electrohydrodynamic forcing (glycocalyx-patch charge and field–flow coupling) to incorporate EHD into the closures for effective advection and dispersion. Experimentally, we will develop refined organ-on-chip platforms with tunable wall charge and co-cultured endothelium–stroma to control EHD conditions *in vitro*, enabling direct quantification of perfusion trends under EHD versus Non-EHD and more rigorous validation of the modeling framework. Together, these advances will broaden biological realism and improve the predictive scope of EHD-aware tumor perfusion modeling.

## Data Availability

The datasets presented in this study can be found in online repositories. The names of the repository/repositories and accession number(s) can be found below: https://figshare.com/s/bcbcae38be369f8ef98b.
