# Supplementary material for: Reduced-order modeling of solute transport within physiologically realistic solid tumor microenvironment
Source: Front Pharmacol. 2026 Mar 10;17:1746751. doi: 10.3389/fphar.2026.1746751 (PMC13008847; doi:10.3389/fphar.2026.1746751)
Supplement: Supplementary file 1 [file DataSheet1.pdf]

# Supplementary Material

## 1 SUPPLEMENTARY DATA - MODEL SENSITIVITY ANALYSIS

### S1. Image-to-scalar mapping and ROI definition

For each paired snapshot (EHD and Non-EHD) associated with a given vessel geometry, we (i) map RGB colors to the scalar concentration index $C_i \in [0, 1]$ using a lookup table extracted from the colorbar image, (ii) automatically detect a region-of-interest (ROI) as the largest contiguous non-white region in each snapshot, and (iii) evaluate all pixelwise metrics on the intersection of the EHD and Non-EHD ROIs to ensure a fair comparison. The per-model mean concentration index $\bar{C}_i$ is computed as the average of $C_i$ over the ROI. All pixel-wise comparisons are computed on the intersection ROI to prevent bias from ROI size differences between EHD and non-EHD snapshots. This conservative choice ensures that any reported EHD enhancement reflects changes within the same spatial support.

### S2. Ensemble robustness of EHD gain

We quantify the EHD gain using the paired per-model difference

$$\Delta \bar{C}_i = \bar{C}_i^{\text{EHD}} - \bar{C}_i^{\text{Non-EHD}}, \tag{S1}$$

and an exceedance-curve separation metric (ABC), defined as the area between exceedance curves $S(\tau) = \mathbb{P}(C_i > \tau)$ integrated over $\tau \in [0, 1]$. In addition, we report a pixelwise dominance fraction (WinRate), defined as the fraction of ROI pixels satisfying $C_i^{\text{EHD}} > C_i^{\text{Non-EHD}}$. Table S1 summarizes all per-model metrics for the 15-model ensemble.

To quantify uncertainty without introducing additional simulations, we perform nonparametric bootstrap resampling over the ensemble (B=10,000). The bootstrap distributions yield confidence intervals for the

**Table S1.** Per-model image-derived concentration index metrics and hemodynamic summary statistics (15-model ensemble).

| Model | $x_f$ ($\mu$m) | $x_f^*$ | $C_i$ (EHD) | $C_i$ (Non-EHD) | $\Delta C_i$ | ABC | WinRate |
|---|---|---|---|---|---|---|---|
| 1 | 25.00000 | 0.00000 | 0.79320 | 0.61851 | 0.17469 | 0.17514 | 0.73405 |
| 2 | 57.14286 | 0.07143 | 0.86088 | 0.60785 | 0.25303 | 0.25363 | 0.76714 |
| 3 | 89.28571 | 0.14286 | 0.81928 | 0.58263 | 0.23665 | 0.23754 | 0.75782 |
| 4 | 121.42857 | 0.21429 | 0.84641 | 0.59477 | 0.25164 | 0.25223 | 0.74878 |
| 5 | 153.57143 | 0.28571 | 0.67457 | 0.49807 | 0.17651 | 0.17932 | 0.77300 |
| 6 | 185.71429 | 0.35714 | 0.73553 | 0.54990 | 0.18563 | 0.18713 | 0.71141 |
| 7 | 217.85714 | 0.42857 | 0.76174 | 0.57989 | 0.18185 | 0.18275 | 0.73491 |
| 8 | 250.00000 | 0.50000 | 0.63955 | 0.55477 | 0.08478 | 0.08658 | 0.73067 |
| 9 | 282.14286 | 0.57143 | 0.86041 | 0.69278 | 0.16763 | 0.16826 | 0.75932 |
| 10 | 314.28571 | 0.64286 | 0.83362 | 0.59547 | 0.23814 | 0.23860 | 0.74186 |
| 11 | 346.42857 | 0.71429 | 0.69048 | 0.57600 | 0.11449 | 0.11531 | 0.73121 |
| 12 | 378.57143 | 0.78571 | 0.63400 | 0.56980 | 0.06420 | 0.06589 | 0.59746 |
| 13 | 410.71429 | 0.85714 | 0.80033 | 0.57977 | 0.22056 | 0.22160 | 0.73350 |
| 14 | 442.85714 | 0.92857 | 0.79889 | 0.57447 | 0.22442 | 0.22574 | 0.74149 |
| 15 | 475.00000 | 1.00000 | 0.79360 | 0.61576 | 0.17784 | 0.17780 | 0.75536 |

**Table S2.** Bootstrap-based robustness and screening sensitivity summary (B=10,000 resamples). Reported intervals are percentile bootstrap 95% CIs. Location screening uses normalized fenestra position $x_f^*$. Prox/mid/dist bins correspond to models 1–5, 6–10, and 11–15.

| Quantity | Estimate | 95% CI (lo) | 95% CI (hi) |
|---|---|---|---|
| Mean $\Delta \bar{C}_i$ | 0.18347 | 0.15376 | 0.21022 |
| Median $\Delta \bar{C}_i$ | 0.18185 | 0.16763 | 0.22442 |
| $\mathbb{P}(\Delta \bar{C}_i > 0)$ | 1.00000 | 1.00000 | 1.00000 |
| Spearman $\rho$ ($\Delta \bar{C}_i$ vs $x_f^*$) | -0.27500 | -0.74292 | 0.30130 |
| $R^2$ (linear: $\Delta \bar{C}_i$ vs $x_f^*$) | 0.08690 | 0.00027 | 0.47900 |
| Spearman $\rho$ (ABC vs $x_f^*$) | -0.31071 | -0.76492 | 0.26868 |
| $R^2$ (linear: ABC vs $x_f^*$) | 0.08808 | 0.00029 | 0.47275 |
| Kruskal–Wallis $p$ (bins: $\Delta \bar{C}_i$) | 0.32628 | – | – |
| Kruskal–Wallis $p$ (bins: ABC) | 0.26448 | – | – |

**Table S3.** Across-ensemble variability (robustness) quantified using coefficient of variation (CV)

| Metric | Mean | Std | CV |
|---|---|---|---|
| $\bar{C}_i$(EHD) | 0.76950 | 0.07736 | 0.10054 |
| $\bar{C}_i$(nonEHD) | 0.58603 | 0.04190 | 0.07150 |
| $\Delta$ Ci | 0.18347 | 0.05824 | 0.31746 |
| ABC | 0.18450 | 0.05798 | 0.31424 |
| WinRate | 0.73453 | 0.04122 | 0.05612 |

**Table S4.** Global screening using rank-based partial correlation coefficients (PRCC) with bootstrap 95% confidence intervals. Inputs: $x_f^*$ and $u_{95}$. Outputs: $\Delta \bar{C}_i$ and ABC.

| output | parameter | PRCC | $CI_{lo}$ | $CI_{hi}$ |
|---|---|---|---|---|
| $\Delta \bar{C}_i$ | $x_f^*$ | -0.43494 | -0.84740 | 0.16737 |
| $\Delta \bar{C}_i$ | $u_95$ | -0.47760 | -0.84392 | 0.14219 |
| ABC | $x_f^*$ | -0.47319 | -0.85664 | 0.09718 |
| ABC | $u_95$ | -0.49189 | -0.84687 | 0.13578 |

mean/median EHD gain and for screening correlations with fenestra location. Table S2 provides a summary of bootstrap-derived estimates and 95% confidence intervals.

## S3. Variability across the 15-model ensemble

We report across-ensemble variability using the coefficient of variation (CV = std/| mean |) for $\bar{C}_i$ (EHD and Non-EHD) and gain metrics (Table S3). CV is used here as a robustness/variability descriptor across the ensemble (not as a variance-based Sobol index). CV is reported to characterize the spread of outcomes across the finite ensemble and to show that the observed gain is not driven by a single outlier model

## S4. Global screening sensitivity (PRCC)

Because additional CFD cases were not performed, we complement the location-ensemble analysis with a rank-based global screening sensitivity method using partial rank correlation coefficients (PRCC). We treat the normalized fenestra location $x_f^*$ and a hemodynamic covariate ($u_{95}$, the 95th percentile velocity) as screening inputs, and evaluate PRCC for $\Delta \bar{C}_i$ and ABC. Bootstrap resampling (B=10,000) provides 95%

confidence intervals. Table S4 reports PRCC values and confidence intervals, and Fig. **??–??** visualize the screening results.

## S5. Sensitivity to fenestra location (variance explained)

We assess how much of the across-ensemble variance is explained by fenestra location by fitting a linear model to $\Delta\bar{C}_i$ and to ABC as functions of $x_f^*$. We report the slope and coefficient of determination ($R^2$) with bootstrap confidence intervals (Table S2). Additionally, we assess differences across proximal/mid/distal location bins using a Kruskal–Wallis test (Table S2). The resulting $R^2$ values are low and the bootstrap intervals are broad, indicating that fenestra location explains only a small fraction of the across-ensemble variance in $\Delta\bar{C}_i$ and ABC; the enhancement persists across the location span rather than being concentrated in one region.

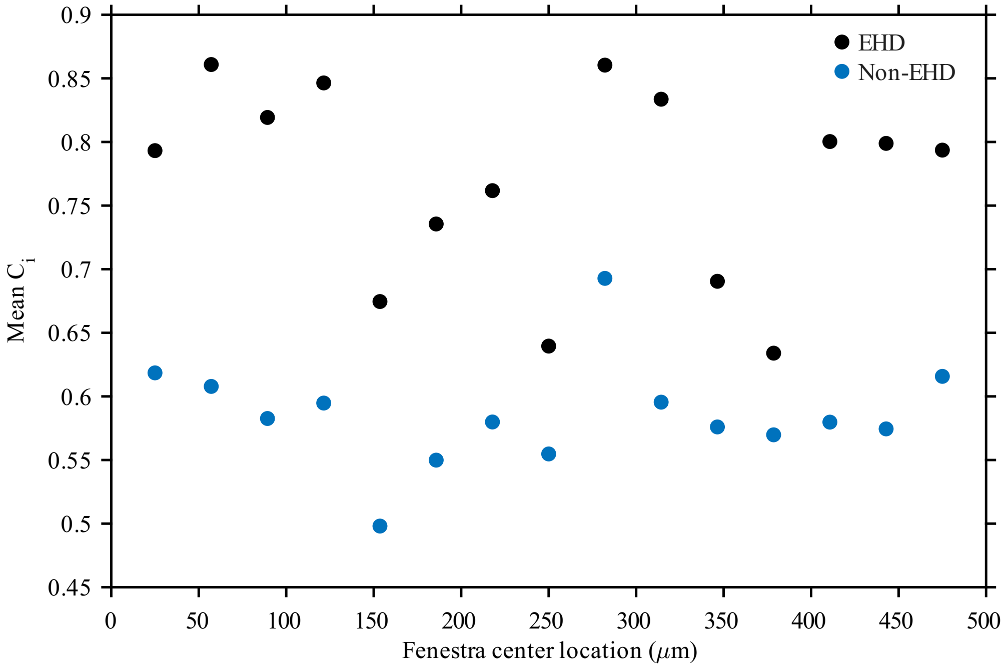


**Figure S1.** Paired comparison of mean concentration index $\bar{C}_i$ in the auto-detected ROI for EHD vs Non-EHD across the 15-model ensemble. The diagonal line indicates equality.

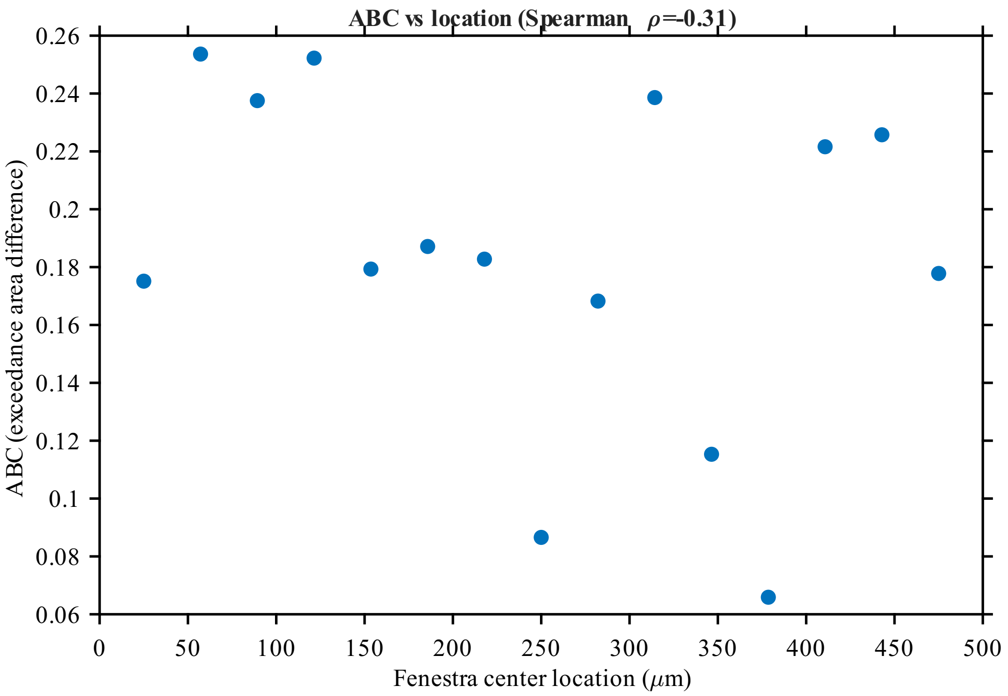


**Figure S2.** Exceedance-area metric (ABC) as a function of fenestra center location for the 15-model ensemble. Each marker corresponds to one model realization, and ABC is computed as the area between the EHD and non-EHD exceedance curves over the concentration index threshold range (positive ABC indicates EHD dominance). The Spearman rank correlation between ABC and normalized location is $\rho = -0.31$, indicating only a weak monotonic dependence on location.

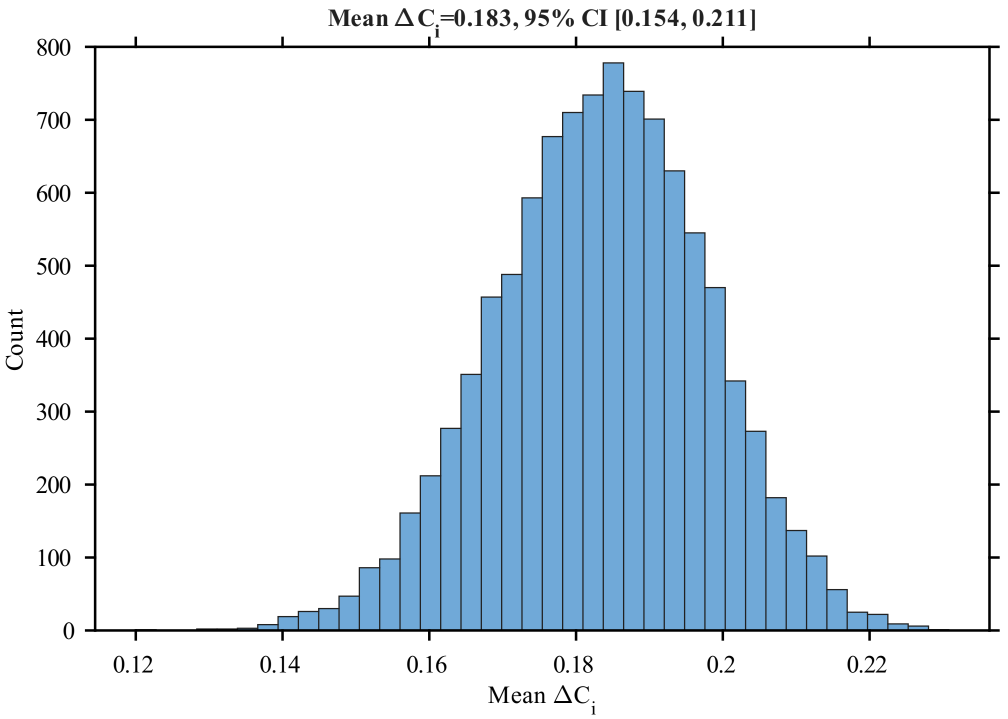


**Figure S3.** Bootstrap distribution of the ensemble-mean concentration-index gain, $\overline{\Delta C_i} = \overline{C_{i,\mathrm{EHD}} - C_{i,\mathrm{nonEHD}}}$, obtained from $B = 10{,}000$ resamples (with replacement) of the 15-model ensemble. The vertical spread reflects sampling uncertainty in the mean gain; the title reports the bootstrap estimate and the two-sided 95% confidence interval.

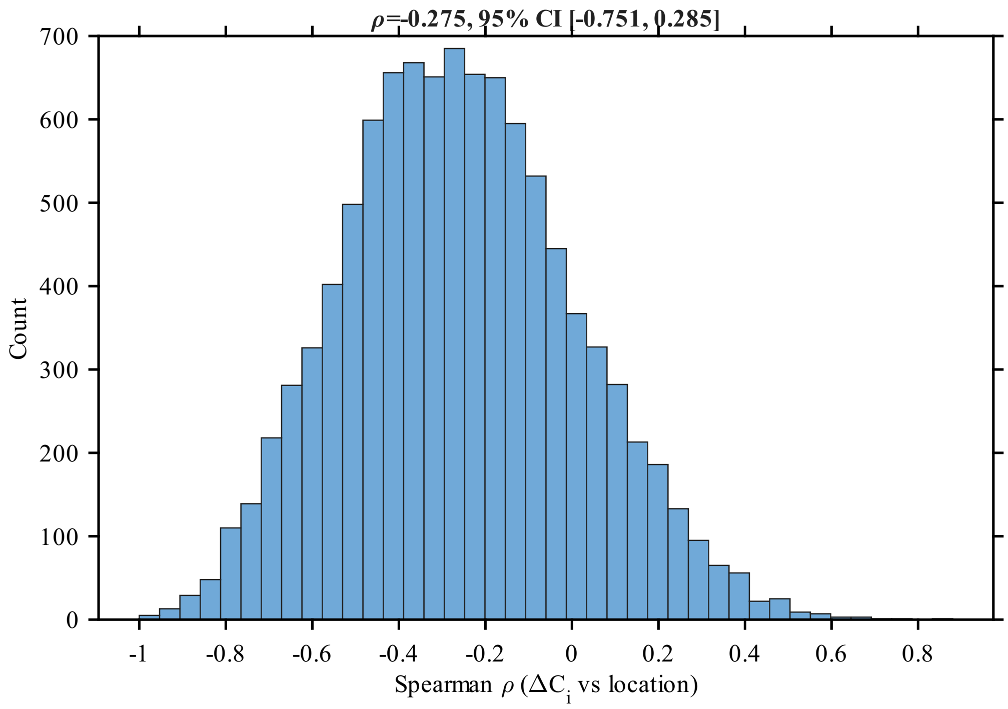


**Figure S4.** Bootstrap distribution of the Spearman rank correlation between fenestra center location ($x_f^*$) and the concentration-index gain $\Delta C_i$ across the 15-model ensemble. Histograms are based on $B = 10{,}000$ bootstrap resamples (with replacement); the reported $\rho$ and two-sided 95% confidence interval summarize the sampling uncertainty in the location–gain association.

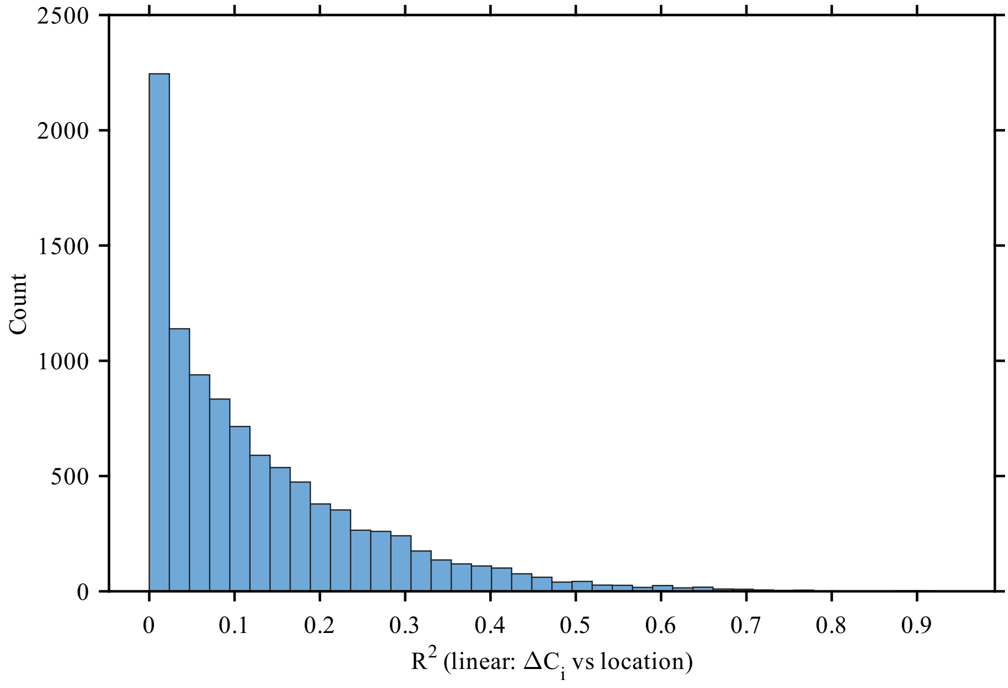


**Figure S5.** Bootstrap distribution of the coefficient of determination ($R^2$) from a linear regression of the gain $\Delta C_i$ on fenestra center location ($x_f^*$) across the 15-model ensemble. Histograms are based on $B = 10{,}000$ bootstrap resamples (with replacement), highlighting the uncertainty in the linear effect size and indicating that location explains only a limited fraction of the across-ensemble variance in $\Delta C_i$.

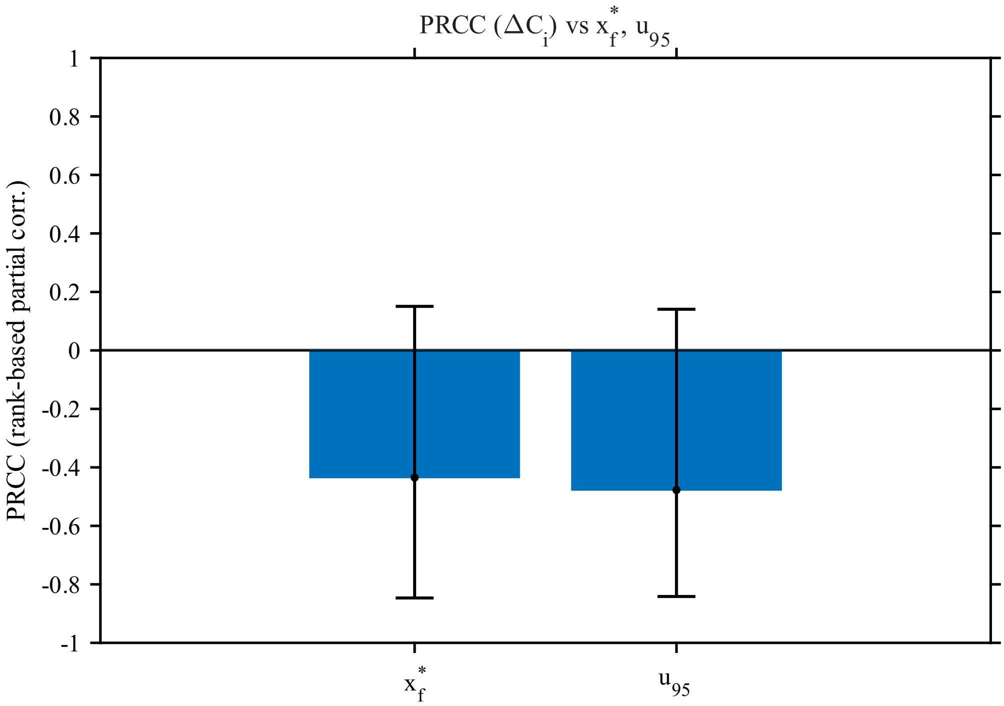


**Figure S6.** PRCC global screening for $\Delta \bar{C}_i$ using inputs $\{x_f^*, u_{95}\}$ with bootstrap 95% confidence intervals..

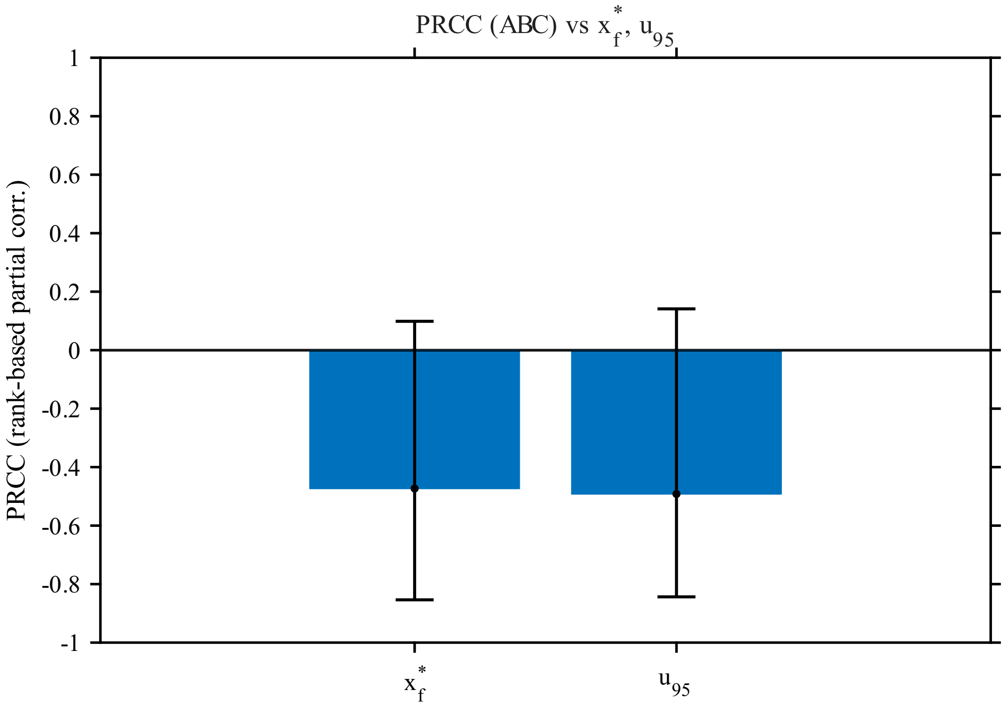


**Figure S7.** PRCC global screening for ABC using inputs $\{x_f^*, u_{95}\}$ with bootstrap 95% confidence intervals.
